# Transcriptional and Functional Analysis of the Effects of Magnolol: Inhibition of Autolysis and Biofilms in *Staphylococcus aureus*

**DOI:** 10.1371/journal.pone.0026833

**Published:** 2011-10-28

**Authors:** Dacheng Wang, Qi Jin, Hua Xiang, Wei Wang, Na Guo, Kaiyu Zhang, Xudong Tang, Rizeng Meng, Haihua Feng, Lihui Liu, Xiaohong Wang, Junchao Liang, Fengge Shen, Mingxun Xing, Xuming Deng, Lu Yu

**Affiliations:** 1 Key Laboratory of Zoonosis, Ministry of Education, Institute of Zoonosis, College of Animal Science and Veterinary Medicine, Jilin University, Changchun, People's Republic of China; 2 Institute of Pathogen Biology, Chinese Academy of Medical Sciences, Beijing, People's Republic of China; 3 Laboratory of Nutrition and Functional Food, Jilin University, Changchun, People's Republic of China; 4 Key Lab for New Drug Research of TCM, Research Institute of Tsinghua University in Shenzhen, Shenzhen, People's Republic of China; 5 School of Public Health, Jilin University, Changchun, People's Republic of China; 6 Department of Infectious Diseases, First Hospital of Jilin University, Changchun, People's Republic of China; 7 College of Veterinary Medicine, Northwest A & F University, Yangling, Shanxi, People's Republic of China; University of Edinburgh, United Kingdom

## Abstract

**Background:**

The targeting of *Staphylococcus aureus* biofilm structures are now gaining interest as an alternative strategy for developing new types of antimicrobial agents. Magnolol (MOL) shows inhibitory activity against *S. aureus* biofilms and Triton X-100-induced autolysis *in vitro*, although there are no data regarding the molecular mechanisms of MOL action in bacteria.

**Methodology/Principal Findings:**

The molecular basis of the markedly reduced autolytic phenotype and biofilm inhibition triggered by MOL were explored using transcriptomic analysis, and the transcription of important genes were verified by real-time RT-PCR. The inhibition of autolysis by MOL was evaluated using quantitative bacteriolytic assays and zymographic analysis, and antibiofilm activity assays and confocal laser scanning microscopy were used to elucidate the inhibition of biofilm formation caused by MOL in 20 clinical isolates or standard strains. The reduction in *cidA*, *atl*, *sle1*, and *lytN* transcript levels following MOL treatment was consistent with the induced expression of their autolytic repressors *lrgA*, *lrgB*, *arlR*, and *sarA*. MOL generally inhibited or reversed the expression of most of the genes involved in biofilm production. The growth of *S. aureus* strain ATCC 25923 in the presence of MOL dose-dependently led to decreases in Triton X-100-induced autolysis, extracellular murein hydrolase activity, and the amount of extracellular DNA (eDNA). MOL may impede biofilm formation by reducing the expression of *cidA*, a murein hydrolase regulator, to inhibit autolysis and eDNA release, or MOL may directly repress biofilm formation.

**Conclusions/Significance:**

MOL shows *in vitro* antimicrobial activity against clinical and standard *S. aureus* strains grown in planktonic and biofilm cultures, suggesting that the structure of MOL may potentially be used as a basis for the development of drugs targeting biofilms.

## Introduction

*Staphylococcus aureus* (*S. aureus*) is one of the most important pathogens in both hospitals and communities and causes numerous diseases in humans, such as endocarditis, osteomyelitis and septicemia [1]. One important virulence trait utilized by *S. aureus* is the ability to form biofilms on damaged tissues and implanted biomaterials [2]. The biofilm structures are inherently resistant to antimicrobial challenge and are difficult to eradicate from the infected host [3]; there is clearly a need for novel antimicrobial agents with new mechanisms of action.

Magnolol (5, 5′-diallyl-2,2′-dihydroxybiphenyl; MOL) is a major component isolated from the stem bark of *Magnolia sp.*, including *Magnolia obovata* and *Magnolia officinalis*, and has been used to treat cough, diarrhea, and allergic rhinitis in Korea, China, and Japan [4]. Magnolol was reported to exhibit potent antibacterial activity against several microorganisms, including methicillin-resistant *S. aureus*, vancomycin-resistant enterococci and *Candida albicans* [5], [6]. Previous report also showed that MOL inhibited biofilm formation by several bacteria, such as *Streptococcus mutans*, *Streptococcus sanguis*, *Actinomyces naeslundii*, *Actinomyces viscosus*, *Enterococcus faecalis*, and *Fusobacterium nucleatum* [7], [8]. However, we found little data in the literature regarding the molecular mechanisms of MOL activity on bacteria grown in biofilm.

Recent reports have shown that autolysis and extracellular DNA (eDNA) release facilitate biofilm formation in *S. aureus in vitro* and *in vivo* [9], [39]. Previous reports have shown that the treatment of *S. aureus* with the protein synthesis inhibitors tetracycline [10] and chloramphenicol [11] or with glycopeptides, including vancomycin [12] and teicoplanin [13], leads to a decrease in autolysis. In contrast, β-lactam antibiotics increase *S. aureus* autolysis [14]. These antibiotics were presumed to function by causing an alteration in proteolytic processing of peptidoglycan hydrolases. The murein hydrolases in staphylococci include N-acetyl muramidase, N-acetyl glucosaminidase, N-acetylmuramyl-L-alanine amidase, endopeptidase and transglycosylases [15]; these enzymes degrade peptidoglycan saccules, resulting in cell lysis. If uncontrolled, these hydrolases can lead to the destruction of the cell wall and cell lysis. Murein hydrolases also have important roles in cell division, including daughter cell separation, peptidoglycan recycling, antibiotic-initiated cell wall lysis and, in some cases, pathogenicity [16]. Thus, we want to investigate whether MOL reduced *S. aureus* biofilm production by inhibiting autolysis *in vitro*.

In this paper, we used quantitative bacteriolytic assays and zymographic analysis to evaluate autolysis inhibition and we used antibiofilm activity assays and microscopy to elucidate the inhibition of biofilm formation caused by MOL in 20 clinical isolates or in standard strains. We explored the molecular basis of the markedly reduced autolytic phenotype and biofilm inhibition triggered by MOL using transcriptomic analysis and we verified the transcription of autolysis-related genes by real-time RT-PCR.

## Results

### Antimicrobial activity of MOL and *S. aureus* growth curves upon exposure to MOL

**Figure 1:**
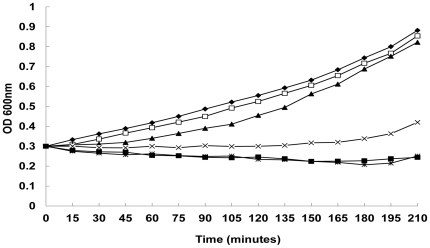
Growth curve for *S. aureus* strain ATCC25923 in the presence of different MOL concentrations at 37°C: ⧫, untreated 0; □, 4 µg/mL; ▴, 8 µg/mL; ×, 16 µg/mL; *, 32 µg/mL; ▪, 64 µg/mL. The data were from a single representative experiment and were reproduced several times.

In this study, the minimum inhibitory concentrations (MICs) of MOL for 20 clinical *S. aureus* strains (15 of which were methicillin-resistant *S. aureus* (MRSA) grown in suspension ranged from 4 to 64 µg/mL, and the MIC_90_ was 32 µg/mL. The minimal bactericidal concentrations (MBCs) of MOL for 20 clinical *S. aureus* strains grown in suspension ranged from 8 to 128 µg/mL, and the MBC_90_ was 128 µg/mL. The MIC and MBC of MOL for ATCC strains 25923 and 29213 grown in suspension were 16 µg/mL and 64 µg/mL, respectively. The results of biofilm identification showed that 8 strains among the 20 clinical *S. aureus* isolates used in this study formed biofilms. The minimum biofilm inhibitory concentration (MBIC) and the minimum biofilm bactericidal concentration (MBBC) of MOL for the 8 biofilm-forming strains grown in biofilm culture were 64 to 128 µg/mL and 512 to 2048 µg/mL, respectively. The MBIC and MBBC of MOL for ATCC 25923 and 29213 grown in biofilm cultures were 64 µg/mL and 512 µg/mL, respectively. These results suggest that MOL is active against *S. aureus* grown in planktonic and biofilm cultures. The growth curve of *S. aureus* ATCC 25923 demonstrated that MOL concentrations of 16, 32 and 64 µg/mL strongly inhibited the growth of planktonic bacteria (Fig. 1).

**Figure 2:**
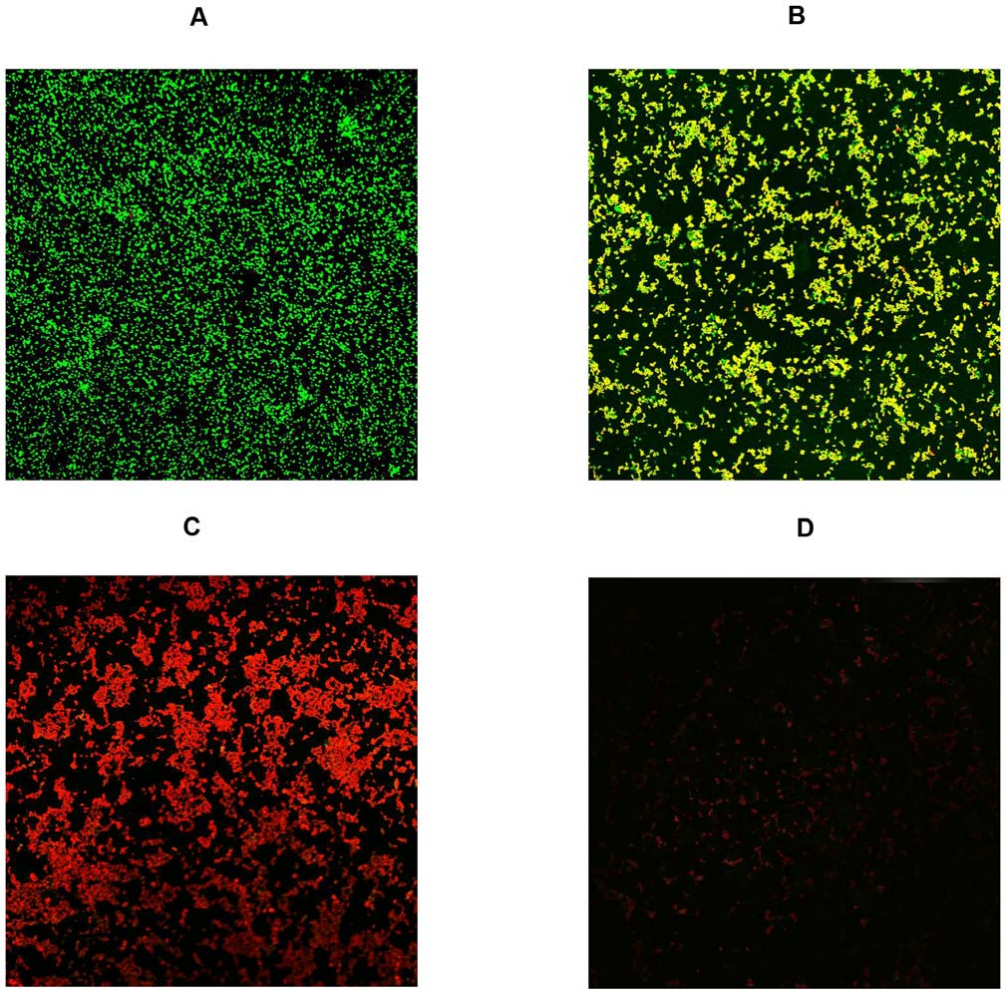
Confocal laser scanning microscopy image of LIVE/DEAD®-stained illustrating the effects of different MOL concentrations on established *S. aureus* ATCC 25923 biofilm formation. Biofilms were formed on coverslides within 48 h at 37°C. Established biofilms were treated with MOL at 128 µg/mL, 256 µg/mL, or 512 µg/mL for 48 h at 37°C. (A) Control (untreated); (B to D) treatment with MOL at 128 µg/mL, 256 µg/mL, or 512 µg/mL respectively. Green, viable cells; Red, dead cells.

The effect of MOL on preexisting biofilms was studied using confocal laser scanning microscopy (CLSM) (Fig. 2). After treatment for 48 h, the control group was chiefly comprised of living bacterial cells (Fig. 2A). Compared with the control group, treatment with 128 µg/mL (2× MBIC) of MOL killed a significant portion of the bacterial population, reduced the number of bacteria present in the biofilm (Fig. 2B), and detached the biofilms. Biofilm bacteria are killed by MOL at concentrations of 256 µg/mL and 512 µg/mL (MBBC), and these concentrations of MOL were also able to detach biofilms (Fig. 2D).

### Overview of transcriptional profiles

GeneChip analysis revealed that a large number of genes (550) were differentially regulated in response to sub-inhibitory concentrations of MOL. Two hundred ten genes showed significant increases and 340 genes showed significant decreases in transcription. The microarray data were submitted to Gene Expression Omnibus (GEO) under the accession number GSE 13236, and a complete list of all genes that were differentially expressed following MOL treatment can be found in the supplementary material (Table S1). We compared the genes that were differentially regulated by MOL treatment with those identified in previous *S. aureus* global transcriptional profiles [17]–[21]; our interest was mainly focused on autolysis-, biofilm- and virulence-associated genes involved in the response to MOL.

We analyzed the expression levels of 77 genes involved in autolysis or related regulators using a microarray (Table S2), and we compared the 106 genes involved in biofilms in previously reported microarray results [22], [23] with counterparts in our result (RNA isolated from planktonic cultures) (Table S3). A small subset of those transcripts was also evaluated by real-time RT-PCR (Table 1).

**Table 1:** Real-time RT-PCR analysis of autolysis and biofilm-related gene expression.

N315ORF	N315gene	N315 description	Fold change ± SD^a^	
			RT-PCR Microarray	
SA0905	*atl*	Bifunctional precursor autolysin Atl	−9.4±3.1	−3.3±0.5
SA0423	*sle1*	N-Acetylmuramyl-L-alanine amidase	−29.2±7.8	−10.0±2.1
SA2329	*cidA*	Hypothetical protein, similar to transcriptional regulator	−6.7±2.3	−3.3±2.3
SA0265	*lytM*	Peptidoglycan hydrolase	−1.7±0.5	−1.4±0.1
SA1090	*lytN*	LytN protein	−1.6±1.0	−1.5±0.2
SA0252	*lrgA*	Murein hydrolase regulator LrgA	+82.6±10.7	+35.0±1.6
SA0253	*lrgB*	Antiholin-like protein LrgB	+80.5±2.8	+30.9±5.5
SA1248	*arlR*	Truncated, putative response regulator ArlR	+4.4±1.1	+2.0±0.2
SA0573	*sarA*	Staphylococcal accessory regulator A	+6.8±2.9	+3.1±0.8
SA2328	*cidB*	Conserved hypothetical protein	+3.4±1.0	+2.3±0.3
SA2327	*cidC*	Pyruvate oxidase	+9.6±1.7	+3.4±0.2
SA1844	*agrA*	Accessory gene regulator A	+10.5±2.8	+3.1±0.6
SAS065	*RNAIII*	Delta haemolysin	+3.4±1.0	+2.0±0.1
SA0251	*lytR*	Two-component response regulator	−3.8±1.9	−2.7±0.2
SA0250	*lytS*	Two-component sensor histidine kinase	−1.9±1.3	−1.6±0.1
SA0641	*mgrA*	Transcriptional regulator MgrA	+1.1±0.7	+1.2±0.1
SA1246	*arlS*	Sensor histidine kinase ArlS	−1.0±1.0	−1.2±0.2
SA2459	*icaA*	Intercellular adhesion protein A	−1.1±0.9	Absent
SA2462	*icaC*	Intercellular adhesion protein C	+1.1±1.2	Absent

a − indicates reduction and + indicates increase.

### Expression levels of autolysis-associated genes following treatment with MOL

Microarray results showed that transcript levels of the main autolysin genes *atl*, *sle1* and *cidA* significantly decreased by a factor of 3.3, 10.9 and 3.3, respectively, and transcript levels of *lytM* and *lytN* were slightly decreased. The expression levels of the negative regulators of autolysis *lrgA*, *lrgB*, *arlR* and *sarA* were significantly increased by 35.0-, 30.9-, 2.0- and 3.1-fold, respectively. Reduced *atl*, *sle1* and *cidA* transcript levels were consistent with the induced expression of their autolytic repressors *lrgA*, *lrgB*, *arlR* and *sarA* in the MOL-treated strain compared with the control strain, which individually or collectively may contribute to the autolysis-inhibited phenotype according to previous studies [24], [25]. Real-time RT-PCR confirmed the decreases in *atl*, *sle1*, *cidA*, *lytM* and *lytN* levels and the increases in *lrgA*, *lrgB*, *arlR* and *sarA* levels (Table 1). Surprisingly, the transcript levels of the autolysin genes *cidBC* were significantly increased. The *cidB* and *cidC* genes are also co-expressed as a transcript separate from *cidABC*, and this *cidBC* transcript is regulated by signals (i.e. sigB) that are independent of *cidABC* regulation [26]. This mechanism could explain why the microarray showed upregulation of *cidB* and *cidC* and downregulation of *cidA*. The microarray data showing changes in *cidBC*, *agrA*, *RNAIII*, *lytR* and *lytS* transcript levels in strain ATCC 25923 exposed to the same concentration of MOL were confirmed by real-time RT-PCR; there were also no significant changes in *mgrA* and *arlS* levels (Table 1). SA0904, which encodes a probable ATL autolysin transcription regulator, and *eprH*, which encodes an endopeptidase resistance factor, were inhibited by 1.6- and 2.2-fold, respectively.

Apart from the major autolysins, SceD and IsaA share a conserved transglycosylase/muramidase domain, and SsaA (SA2093) and four structurally related proteins (SA0620, SA0710, SA2097 and SA2353) all share a common CHAP (cysteine, histidine-dependent amidohydrolase/peptidase) domain. All of these proteins have amino-terminal signal sequences, indicating that they are likely to be exported and/or targeted to the cell wall or membrane [27]. In this study, *isaA* (SA2356), *sceD* (SA1898), *ssaA* (SA2093), SA0620, SA2097 and SA2353 were significantly inhibited by MOL treatment,, but SA0710 was not significantly regulated by MOL. The expression levels of *sspA*, *scpA*, *scpB* and *htrA* were decreased >2-fold, and those of *sspB*, *sspC*, and *aur* were decreased >1.5-fold. *S. aureus* V8 protease, the major serine protease in *S. aureus*, is encoded by *sspA* [28]. A loss of serine protease function has been shown to result in a pleiotropic effect on the profile of secreted proteins, including autolytic activity and proteolytic maturation of the cysteine protease SspB [29]. The decreased levels of *sspA* may also affect the processing of autolysins such as Atl, resulting in altered autolytic activity [16]. The genes *fmtA*, *fmtB*, *tagO*, and *sarV* showed no significant changes in transcript levels, although these genes were recently described as positive regulators of autolysis [30]. The cell division proteins *ftsA*, *ftsZ*, and *scdA* showed marginal (<30%) changes in transcript levels. The transcript level of *fmtC* was decreased by 2.5-fold, but its impact on autolysis is ambiguous [29].

Additionally, *dltABCD* transcript levels were upregulated by more than 2.5-fold in the strain treated with MOL. The complete absence of D-alanine esters in teichoic acids was reported to simultaneously increase the rate of autolysis. Previous transcriptional profiling revealed decreased transcription of the teichoic acid gene *tagB*, and this finding was suggested to be responsible for the reduced whole-cell autolysis that was observed previously [20]. The reduced autolysis phenotype observed in vancomycin-resistant JH9 isolates has been attributed to changes in cell wall teichoic acids [31]. The *dlt* operon of Gram-positive bacteria consists of four genes (*dltABCD*) that catalyze the D-alanyl esterification of glycerolphosphate and ribitol phosphate teichoic acids; the D-alanyl esterification of teichoic acids plays an important role in host-pathogen interactions of *S. aureus* [32]. It was shown that *dltABCD* transcript levels were consistently reduced by about 2-fold in a teicoplanin-resistant strain compared to a teicoplanin-susceptible strain [21].

### Expression levels of biofilm-associated genes following treatment with MOL

We compared the expression of 106 genes involved in biofilms in our microarray with the previously reported results from biofilm cultures [22], [23] (Table S3). The expression levels of 12 genes were significantly induced, but the values of induction were significantly lower than those reported for bacteria grown in biofilms. Twenty-four genes were significantly repressed to an extent similar to bacteria grown in the exponential and stationary phase of biofilm culture, 12 genes were significantly inhibited and 16 genes were significantly induced in a different direction from those in bacteria grown in the biofilm phase. Thirty-eight genes showed no significant expression upon exposure to MOL, and 4 genes were absent from the microarray.

A previous study showed that the expression of 48 genes was enhanced at least two-fold in a biofilm compared to both exponential- and stationary-phase planktonic cultures, of which only 12 genes were significantly induced by MOL. However, the values of induction were significantly lower than those of bacteria grown in biofilm, including the genes *arcABC*, which encode the arginine deiminase cluster *arcD*, which encodes an arginine/ornithine transporter that catalyzes the uptake of arginine and concomitant export of ornithine; and 8 genes encoding the urease operon (ure; N315-SA2081-2088). Of considerable note, genes encoding a potassium-specific transport system (*kdp*; N315-SA1879-SA1881) and the pyrimidine biosynthesis operon (*pyr*; N315-SA1041-SA1049) were significantly repressed or not significantly affected by MOL. Eighty-four genes whose expression was reduced by a factor of at least 2 in biofilms compared with both planktonic growth conditions [22], including an oligopeptide transport system (*opp*; N315-SA0845-SA0848) and the genes responsible for purine biosynthesis (*pur*; N315-SA0920-SA0925), were significantly induced or not significantly affected by MOL. Twenty-four genes were significantly repressed to an extent similar to those in bacteria grown in the exponential- and stationary-phase of biofilm culture; most of these genes encode hypothetical or conserved hypothetical proteins with no known function. The gene *spa*, which encodes protein A and was markedly downregulated in biofilms (60 to 139 times higher in the exponential-phase cultures and 12 to 27 times higher in the stationary-phase cultures), was induced 1.7-fold. The above results suggests that MOL generally inhibits or reverses the expression of most of the genes involved in biofilm, which may be one of the mechanisms through which MOL inhibits this phenotype.

### MOL decreases Triton X-100-induced autolysis

**Figure 3:**
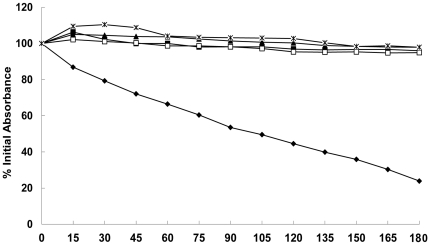
Effect of MOL on Triton X-100-induced autolysis. Triton X-100 was used to stimulate autolysis in *S. aureus* ATCC 25923 cells grown in the absence or presence of various concentrations of MOL. The four concentration investigated were *, 2× MIC; ▴, 1× MIC; △, 1/2× MIC; □, 1/4× MIC; ⧫, untreated. The data were from a single representative experiment and were reproduced several times.

*S. aureus* ATCC 25923 was treated with various concentrations of MOL, and the rate of autolysis induced by the non-ionic detergent Triton X-100 was compared with that of an untreated control culture (Fig. 3). After 180 min, the OD_600_ values of the cells treated with 1/4× MIC, 1/2× MIC, 1× MIC, and 2× MIC MOL and the control cultures were 94.9%, 96.0%, 97.9%, 98.1%, and 23.9% of the initial value, respectively. This finding indicates that Triton X-100-induced autolysis in *S. aureus* ATCC 25923 was inhibited by MOL.

### MOL alters the bacterial peptidoglycan hydrolase profile

Autolysins involved in the tightly controlled maintenance of cell wall integrity during cell division are classified according to their specific cleavage types and include *N*-acetylmuramidases, *N*-acetylglucosaminidases, *N*-acetylmuramyl-L-alanine amidases, endopeptidases and transglycosylases [16]. The major autolysis gene (*atl*) of *S. aureus* encodes a 63-kDa amidase and a 54-kDa glucosaminidase after processing [33]. Other autolytic genes include *sle1*, which encodes a 32-kDa N-acetylmuramyl-L-alanine amidase that is distinct from *atl* [34]; *lytM*, which encodes a glycylglycine endopeptidase [35]; and *lytN*, which possibly encodes a muramidase [36]. A previous study has demonstrated that there were two higher molecular weight hydrolytic bands that likely represented native pro-Atl (134 kDa) and an early 113-kDa Atl processing intermediate [37].

**Figure 4:**
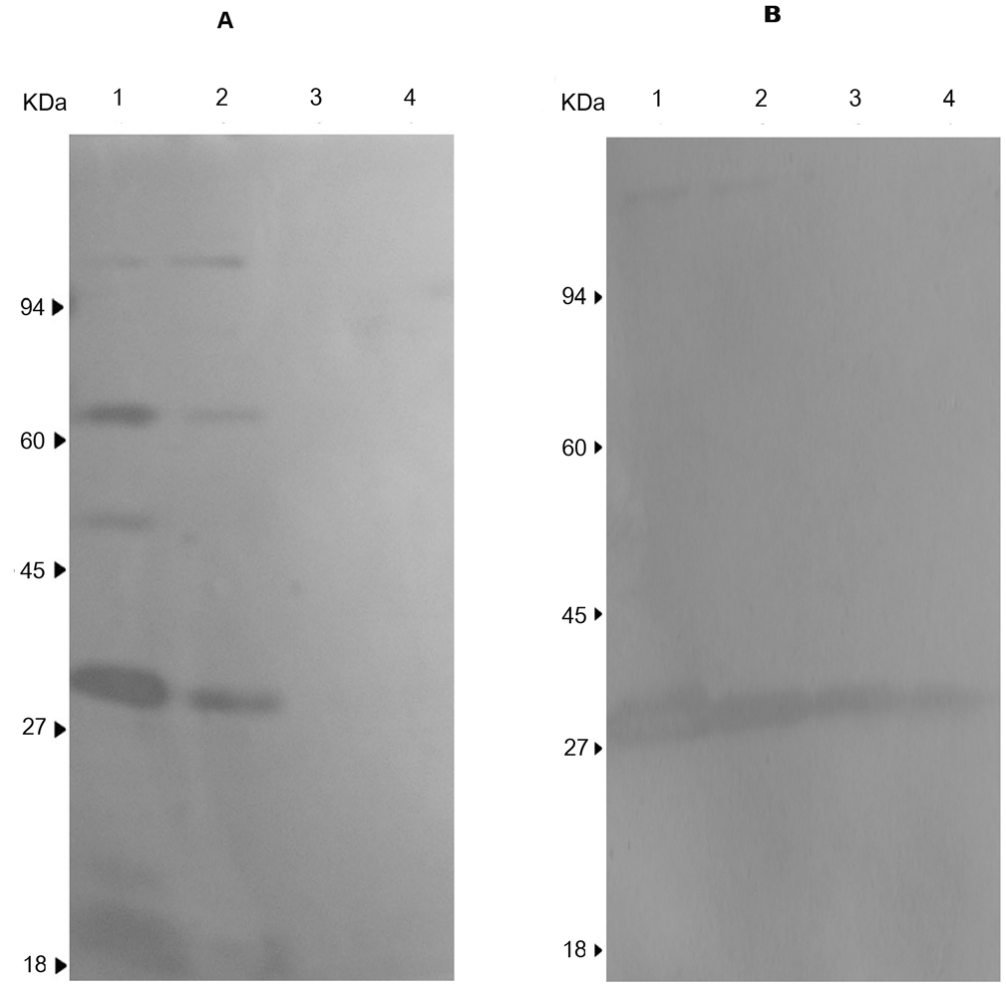
Zymographic analysis of bacteriolytic hydrolase activities of *S. aureus* ATCC 25923 cells treated with MOL. LiCl (A) and SDS (B) autolysin extracts containing *S. aureus* ATCC 25923 cell walls treated with various concentrations of MOL. Lane 1, no MOL treatment; lane 2, 1/4× MIC MOL; lane 3, 1/2× MIC MOL; lane 4, 1× MIC MOL. Molecular size markers are indicated on the left. The data shown are from a single representative experiment and were reproduced several times.

We determined the peptidoglycan hydrolase profiles to investigate the mechanism of MOL autolysis inhibition. Figure 4A shows the results of SDS-polyacrylamide gel electrophoresis of autolysin-digested cell walls after treatment with MOL; untreated ATCC 25923 cells were used as a control. In the LiCl extracts, we found that the 1/4× MIC MOL treatment led to a significant decrease in the 32-kDa and 63-kDa proteins and a complete loss of the approximately 54-kDa protein; 1/2× MIC, 1× MIC MOL and 2× MIC (data not shown) led to the complete loss of the proteins having estimated molecular masses of 113, 63, 54 and 32 kDa. In the SDS extracts, we found that the 1/4× MIC MOL treatment led to a slight inhibition of the expression of the proteins with an estimated molecular mass of 32 kDa; 1/2× MIC, 1× MIC MOL and 2× MIC MOL (data not shown) led to a significant decrease in the 32-kDa species and the complete loss of the proteins having estimated molecular masses of 113 and 134 kDa (Fig. 4B). These results indicate that changes in expression or posttranscriptional or proteolytic processing of *Atl* and other autolysin genes occurred after MOL treatment.

**Figure 5:**
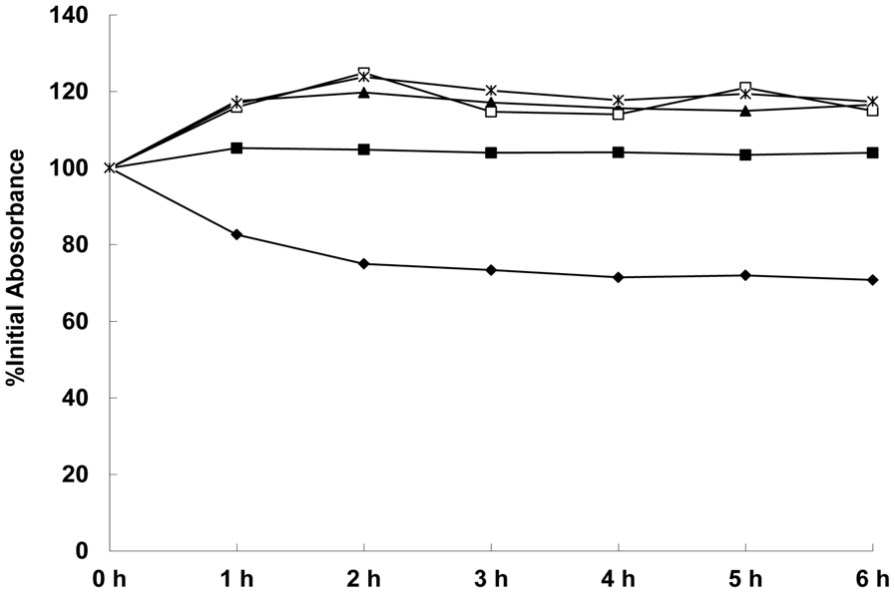
Quantitative analysis of extracellular bacteriolytic hydrolase activities in *S. aureus* ATCC 25923 cells treated with various concentration of MOL. The four concentration investigated were *, 2× MIC; □, 1× MIC; ▴, 1/2× MIC; ▪, 1/4× MIC; ⧫, untreated. The data were from a single representative experiment and were reproduced several times.

To extend the zymographic analysis, quantitative bacteriolytic assays of extracellular *S. aureus* ATCC 25923 proteins against lyophilized *S. aureus* suspensions were performed (Fig. 5). After a 6 hr MOL treatment, the extracellular murein hydrolases of *S. aureus* did not decrease the turbidity of *S. aureus* cells as much as untreated cells. MOL treatments of 1/4× MIC, 1/2× MIC, 1× MIC and 2× MIC of the extracellular murein hydrolases caused 33.3%, 45.9%, 44.3% and 46.7% decreases in turbidity, respectively, compared to that in the control *S. aureus* cells. These results, along with the zymographic analysis, demonstrate that MOL treatment results in a reduction of both cell-wall associated and extracellular murein hydrolase activity.

### Role of MOL on DNA release by *S. aureus*

**Figure 6:**
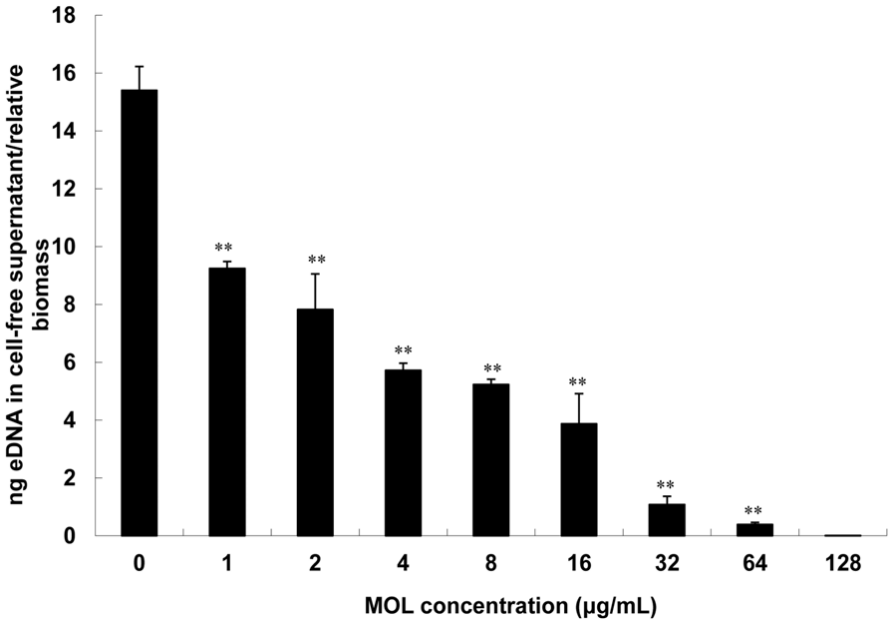
Role of MOL on DNA release of *S. aureus*. The amount of eDNA in the cell-free supernatants from the *S. aureus* strain ATCC 25923 biofilms treated with MOL at concentration of 1/16∼8× MIC was measured by spectrophotometry. The values are expressed as nanogram of eDNA per relative biofilm biomass (OD600). Values represent the mean ± SD for three independent experiments. ** represents *p*<0.01.

The amounts of eDNA in the cell-free supernatants from the biofilms was quantified by spectrophotometry and reported as eDNA per relative biomass to account for the amount of bacteria present in the biofilm treated by different concentration of MOL. As shown in Fig. 6, treatment with MOL resulted in a dose-dependent decrease in the amount of eDNA from the cell-free supernatants in *S. aureus* ATCC 25923. Compared with the level in cultures of control strain (without MOL treatment), cultures with more than 1/8× MIC of MOL displayed a significant reduction in the amount of eDNA for strains ATCC 25923 (*p*<0.01); in the cultures with more than 8× MIC of MOL, little or no eDNA could be detected in strains ATCC 25923.

## Discussion

In *S. aureus*, cell death and lysis are controlled by the *cid* and *lrg* operons, the products of which are reported to function as holins and antiholins, respectively, and may serve as molecular control elements for bacterial cell lysis [38]. Recent findings have shown that the *S. aureus cidA* gene promoted cell lysis during biofilm development [39], [9], whereas the *lrg* operon, as an inhibitor of lysis [37], inhibited cell lysis. These studies demonstrated that *cidA*-controlled cell lysis-released genomic DNA is an important structural component of *S. aureus* biofilms [9]. Indeed, eDNA is an essential matrix molecule that is produced during biofilm development by many bacterial species, including *P. aeruginosa* and *S. aureus* [40]. In the present study, the microarray results showed that MOL significantly inhibited *cidA* expression and significantly induced *lrgAB* expression, which is consistent with the phenotype of autolysis inhibition upon exposure to MOL. In the aforementioned results, MOL treatment also inhibited the expression of other autolysis-associated genes as follows: the autolysin genes *atl*, *sle1*, *lytM* and *lytN*; the probable ATL autolysin transcription regulator SA0904; the endopeptidase resistance factor *eprH*; the amino-terminal signal sequence group genes *isaA* (SA2356), *sceD* (SA1898), *ssaA* (SA2093), SA0620, SA2097 and SA2353; and *sspABC*, *scpAB*, *htrA*, *fmtC* and *aur*. In addition, MOL treatment inhibited the expression of major cell wall biosynthesis genes, including *pgcA*, *gtaB*, *fmhA*, *lytH*, SA2354, *tcaB drp35*, *rsbU*, and *purABCKERQ*. Simultaneously, MOL treatment also increased the expression of the negative regulators of autolysis *arlR* and *sarA*. As mentioned above, MOL diminished the amounts of eDNA from *S. aureus* in a dose-dependent manner, with MOL at concentration of more than the MBIC almost completely eliminating eDNA from the tested strains. Thus, we concluded that the phenotype of biofilm inhibition by MOL treatment might be due to an abolishment or reduction of genomic DNA release, leading to a decreased biofilm development. Noteworthily, there is a significant effect of MOL at concentarion of more than the MIC on planktonic growth, as showed in Figure 1, we presume the decrease in planktonic cells is due to inhibition of proper peptidoglycan processing, although MOL at this concentarion repressed biofilm development due to loss of eDNA.

Moreover, comparing our microarray results (RNA isolated from planktonic cultures) for the genes involved in biofilm production with those identified in previous reports [22], [23] (Table S3), we found that MOL widely inhibited or reversed the expression of genes involved in biofilms, suggesting that MOL may also directly inhibit biofilm formation. An important component of many *S. epidermidis* biofilms is the polysaccharide intercellular adhesin PIA, also called polymeric *N*-acetylglucosamine (PNAG), which is synthesized by the *icaADBC*-encoded proteins. PIA is also produced by *S. aureus*, and the *ica* operon appears to be present in virtually all *S. aureus* strains [41]. Likewise, eDNA is found in *S. aureus* biofilms and contributes to the strength of the biofilm matrix [42]. In this study, the microarray results showed that *icaB* was not significantly affected (1.1-fold) upon exposure to MOL, whereas *icaR*, a negative regulator of *icaADBC*, was significantly induced 3.6-fold by MOL. Because *icaACD* genes were not printed on the microarray, we assayed the expression of these genes by real-time RT-PCR and showed that these genes were also not significantly regulated by exposure to MOL. This result may explain why *ica* expression is associated with the initial colonization of *Staphylococcus* in a foreign body infection model but not with bacterial persistence [43].

In this report, MOL inhibited *S. aureus* autolysis and biofilms development and also simultaneously affected the transcription of virulence factors at the same time. Thirty-five genes were downregulated, including *plc*, *sei*, *yent1*, *yent2*, *sem*, *seo*, *fnb*, *geh*, *htrA*, *sspA*, SA1725, *sdrD*, SA1000, SA1003, SA0746, *isaA*, *isdA*, SA0276, *sbi*, SA1751, SA1752, SA1898, SA0276, SA0610, SA1429, *lpl1*, *lpl2*, *lpl7* and *lpl8*. The upregulated virulence factor genes include *clfA*, SA0102, SA2006, and *hlgA*. In *S. aureus*, the regulation of autolytic activity is generally complex and may occur at a number of different levels, including cell wall composition, autolysin enzymatic activity, posttranslational processing of autolysins and autolysin gene transcription [44]. Virulence factor regulation by *S. aureus* is modulated by at least seven two-component systems (TCSs) (ArlRS, SaeRS, AgrAC, SrrAB, LytRS, YycFG, and VraRS), the DNA-binding protein SarA, the SarA family (SarS, SarR, SarU, SarT, SarV, MgrA, and TcaR) and an alternative sigma factor (B). In addition, Rot (a SarA homologue) and agr have opposing effects on select target genes. The two-component system *lytSR* is involved in the regulation of peptidoglycan hydrolases, and *lrgA* and *lrgB* are positively regulated by *lytSR* [45]. Recent reports have revealed the downregulation of *lytSR* and *lrgAB* by ArlRS [18]. A recent report has also indicated that the ClpP proteases strongly influence virulence, stress response and physiology in *S. aureus* [17]; the *clpP* gene was induced 1.4-fold in this study. We also observed that the expression levels of *agrA*, *arlR*, *vraR*, *vraS*, *RNAIII*, and *SarA* were upregulated >1.5-fold, whereas *saeR*, *saeS*, *srrB*, *sarS*, *sigB*, *lrgA*, *lrgB*, *lytS*, and *lytR* expression levels were downregulated >1.5-fold and *arlS*, *agrC*, *srrA*, *yycF*, *yycG*, *sarR*, *sarU*, *sarT*, *sarV*, *mgrA*, *clpP*, and *tcaR* expression levels were slightly increased. This finding suggests that the differential expression of autolysis genes and virulence factors may be coordinately regulated by these two-component signal transduction systems and that the transcription regulators that they share may be sensitive to MOL. Resch et al. suggested that processes involved in cell wall synthesis and other distinct physiological activities of the cell, rather than toxins and other virulence factors, play a crucial role in biofilm persistence [23].

In summary, MOL may inhibit biofilm formation by reducing the expression of the murein hydrolase regulator *cidA*, which then inhibits autolysis and extracellular DNA (eDNA) release to impede biofilm formation; MOL may also directly repress biofilm formation. Additionally, the decrease in lysis caused by MOL is accompanied by peptidoglycan hydrolase profile changes and transcriptional changes in the related autolysis regulators, presumably due to an alteration in proteolytic processing. The expression levels of some important virulence factors were also decreased. All of these events may be important to the mode of action of MOL.

## Materials and Methods

### Bacterial strains and materials

The *S. aureus* strains consisted of 20 clinical *S. aureus* isolates obtained from the First Hospital of Jilin University that showed different antimicrobial susceptibility patterns and 2 standard strains, ATCC 25923 and ATCC 29213, that were capable of biofilm formation *in vitro* [46] (obtained from the China Medical Culture Collection Center; CMCC). Mueller–Hinton agar (MHA) and Mueller–Hinton broth (MHB) was purchased from Oxoid (Basingstoke, UK). Modified Letheen broth (Becton, Dickinson and Co., France) was prepared and sterilized according to the manufacturer's instructions with the addition of 1% (w/v) bacteriological agar (agar no. 1) (Oxoid). MOL was purchased from CMCC, and stock solutions of varying concentrations were prepared in dimethyl sulfoxide (DMSO, Sigma-Aldrich).

### Determination of MICs and MBCs of MOL for *S. aureus* in suspension

The MICs of MOL against the 22 *S. aureus* strains mentioned above were determined in triplicate by broth microdilution or macrodilution using serial two-fold dilutions in MHB according to standard NCCLS procedures [47]. The MIC was defined as the lowest concentration at which no visible growth was observed. The minimum concentration of MOL that inhibited 90% of the tested isolates was defined as the MIC90. The MBCs were determined by transferring the total volume (200 µL) from each of the clear wells into duplicate plates and mixing with 20 mL of cooled molten MHA, which was then allowed to set. Plates were incubated in air at 37°C for 24 h. The MBC was defined as the first plate yielding no growth [48]. The assay was performed in triplicate.

### Establishment of *S. aureus* biofilm

Confirmation of biofilm production by *S. aureus* strains was performed by applying Alcian Blue stain to each of the wells [49]. To confirm slime production, microorganisms were cultured on Congo Red agar [50]. Bacterial biofilms were prepared by aliquoting 200 mL of bacterial suspension containing 1×10^5^ CFU/mL into the wells of white-walled, clear-bottom, tissue culture-treated 96-well microtiter plates. Empty wells were reserved for use as negative controls. Microtiter plates were then incubated in air for 48 h at 37°C.

### Determination of MBIC and MBBC of MOL for *S. aureus* in biofilms

The MBIC and MBBC of MOL against standard *S. aureus* strains ATCC 25923 and ATCC 29213 and 8 clinical isolates (capable of biofilm formation) were tested [48]. Wells containing biofilms were gently washed with 250 mL of PBS to remove any unbound cells. Serial double-dilutions of MOL were prepared in either MHB (0.125–2048 µg/mL). To triplicate wells, 100 mL of MHB was added to 100 mL of antimicrobial agent in decreasing concentrations across the rows. After 24 h of incubation in air at 37°C, the antimicrobial agents were removed, and the wells were washed once with PBS. PBS (250 mL) was added to each well, and the plate was sonicated at 50 Hz in a water bath for 30 min at room temperature. Biofilms were recovered from the wells by scraping and washing [51], and the entire contents of the well (250 mL) were mixed with molten Letheen broth containing 1% (w/v) agar no. 1 and cooled to 50°C. Following incubation of the set plates in air at 37°C for 24 h, the MIC was determined as the lowest concentration to show growth below or equal to that of the control (biofilm in saline). The MBBC was defined as the lowest concentration of the antibiotic that killed 99.9% of bacterial cells within the biofilm [52].

### CLSM

Images of biofilms were collected for control cells and for cells treated with MOL at concentrations of 64 µg/mL (MBIC), 256 µg/mL, or 512 µg/mL. Aliquots (2 ml) of MHB-diluted overnight culture were used to grow biofilms on coverslips in 6-well dishes for 48 h. The coverslides were then washed carefully with PBS, moved to a new plate and treated for 48 h with MOL. The coverslides were washed again with PBS and stained with a LIVE/DEAD BacLight Bacterial Viability kit (Invitrogen Molecular Probes, Eugene, OR, USA) following the manufacturer's instructions. CLSM images were collected using an Olympus FV1000 confocal laser scanning microscope (Olympus, Tokyo, Japan) with a 60× objective lens. For detection of SYTO 9 (green channel), we used 488 nm excitation and 520 nm emission filter settings. For PI detection (red channel), we used 543 nm excitation and 572 nm emission filter settings. Image analyses and export were performed in using Fluoview version 1.7.3.0 software.

### Growth curves

*S. aureus* strain ATCC 25923 was grown to a 600 nm optical density of 0.3 in MHB II, and 100-mL aliquots were then distributed into five 500-mL Erlenmeyer flasks. MOL (dissolved in DMSO) was added to four of the cultures to obtain final concentrations of 1/4× MIC (4 µg/mL), 1/2× MIC (8 µg/mL), 1× MIC (16 µg/mL) and 2× MIC (32 µg/mL). The final DMSO concentration for all conditions was 1% (vol/vol). The control culture was supplemented with 1% DMSO alone. The cultures were incubated, and cell growth was monitored spectrophotometrically (600 nm optical density was recorded at 15-min intervals).

### Treatment with MOL for microarray analysis

The *S. aureus* strain ATCC 25923 was grown overnight at 200 rpm in a rotary shaker at 37°C in 10 mL of MHB II. Two 250-mL Erlenmeyer flasks, each of which contained 100 mL of MHB II, were inoculated with an overnight culture to an initial OD_600_ of 0.05. The bacteria were grown at 37°C at 200 rpm to an OD_600_ of 0.3. Subsequently, 500 µL of a 1600 µg/mL MOL stock solution, prepared in dimethyl sulfoxide (DMSO), were added to the experimental culture to yield a final concentration of 1/2× MIC (8 µg/mL). The other culture, which contained 1% (vol/vol) DMSO without MOL, was used as the control. The final concentration of DMSO in each culture was 1% (vol/vol); moreover, this amount of DMSO did not alter the pH of the medium. Both experimental and control suspensions were further incubated for 45 min at 37°C, after which the RNA was isolated. Three independent experiments were carried out.

### RNA isolation and cDNA labeling

Immediately before harvesting the bacterial cells for RNA isolation, the samples were treated with the RNA protect Bacteria Reagent (QIAGEN, Inc., Valencia, CA) to minimize RNA degradation. The cells were collected by centrifugation and stored at −80°C. RNA extraction was carried out using the RNeasy Mini kit (QIAGEN) according to the manufacturer's instructions. Contaminating DNA was removed by treatment with RNase-free DNase I (10 U/40 µg total bacterial RNA) at 37°C for 20 min. RNA was then repurified with an RNeasy Mini column (QIAGEN). RNA quality was monitored by agarose gel electrophoresis, and RNA quantity was measured by UV spectrophotometry. RNA was reverse-transcribed into cDNA, which was subsequently labeled [53].

### GeneChip hybridization and analysis

Labeled cDNAs from independent RNA preparations were hybridized to six separate GeneChip *S. aureus* genome arrays according to the manufacturer's instructions for antisense prokaryotic arrays (Affymetrix, Inc.). The GeneChip *S. aureus* genome array (antisense) (Affymetrix, Inc., Santa Clara, CA) has been described in detail in previous studies [53] and includes N315, Mu50, NCTC 8325, and COL. The array contains probe sets for over 3,300 *S. aureus* ORFs and over 4,800 intergenic regions. A total of 1.5 µg of labeled material was hybridized to each GeneChip for 16 h at 45°C [53]. After hybridization, the washing and staining of arrays were performed using the GeneChip® Fluidics Station 450 and scanned with the Affymetrix GeneChip Scanner 3000.

The images were processed with Microarray Analysis Suite 5.0 (Affymetrix) software. The raw data from the array scans were normalized by median-centering genes for each array and were log transformed. Expressed genes were identified using Affymetrix GeneChip Operating Software (GCOS, Ver.1.0), which uses statistical criteria to generate a “present” or “absent” call for the genes represented by each probe set on the array. Additionally, genes with “absent” scores were filtered out, and the remaining genes were analyzed. To identify the genes that were differentially expressed in MOL-treated samples compared with control samples, the Significance Analysis of Microarrays (SAM) software (http://www-stat.stanford.edu/~tibs/SAM/index.html) [54] was used. To select the differentially expressed genes, we used threshold values of ≥1.5- and ≤−1.5-fold change between three MOL treatment samples and three control samples; the FDR significance level was <5%.

### Quantitative real-time RT-PCR

Quantitative real-time reverse transcription (RT)-PCR was used to verify the microarray results. Aliquots of the RNA preparations from the MOL-treated and control samples used in the microarray experiments were saved for quantitative real-time RT-PCR follow-up studies. Quantitative real-time PCR was performed in triplicate using the 7000 Sequence Detection System (Applied Biosystems, Foster City, CA, USA) according to a previously described procedure [53]. The cDNA was subjected to real-time PCR using the primer pairs listed in Table S4.

### Triton X-100-induced autolysis assays

The *S. aureus* strain ATCC 25923 was grown to a 600 nm optical density (OD_600_) of 0.3, at which time MOL was added to the cultures, typically at concentrations of 1/4× MIC, 1/2× MIC, 1× MIC, 2× MIC, or 4× MIC. Growth with shaking at 37°C was allowed to continue until the OD_580_ reached 0.7 in control cultures. The MOL-treated cultures were incubated for the same length of time as the control cultures. The cells were harvested by centrifugation and washed once by resuspension in cold distilled water. The cell pellet was resuspended to an OD_600_ of 1.0 in 0.05 M Tris–HCl, pH 7.0 containing 0.05% (v/v) Triton X-100. The cell suspension was then incubated at 30°C with shaking, and the OD_580_ was determined at various intervals [55].

### Autolytic enzyme extracts

Cultures of *S. aureus* ATCC 25923 were grown to mid-exponential phase in 250 mL of TSB at 37°C with aeration, at which time MOL was added to the cultures, typically at concentrations of 1/4× MIC, 1/2× MIC, 1× MIC or 2× MIC. The cultures were incubated with shaking at 37°C for another 30 min, after which they were rapidly chilled, harvested by centrifugation, washed once in ice-cold 50 mM Tris-Cl, pH 7.5, and extracted with 250 µL of 4% sodium dodecyl sulfate (SDS) at room temperature or 3 M LiCl at 4°C for 30 min with stirring. Protein concentrations were determined using the BCA protein assay kit (Pierce, Rockford, IL) with bovine serum albumin as the standard [12].

### Preparation of crude cell walls

Cultures of *S. aureus* ATCC 25923 were grown in TSB to mid-logarithmic phase at 37°C with aeration, after which they were rapidly chilled, harvested by centrifugation and resuspended in boiling 8% SDS. After boiling for 30 min, the samples were washed in distilled water to remove the SDS, mechanically disrupted, washed again and lyophilized.

### Bacteriolytic enzyme profiles after SDS-polyacrylamide gel electrophoresis

The separation of proteins was carried out using the technique of Laemmli [56]. Resolving gels (7.5% acrylamide–0.2% bisacrylamide) contained crude cell walls (1 mg dry weight per mL). Samples were separated by electrophoresis at a constant current of 20 mA at room temperature until the blue dye reached the bottom of the separating gel. The visualization of bacteriolytic enzymes was carried out as follows: the gels were initially washed in distilled water four times for 15 min each, washed in buffer composed of 50 mM Tris-Cl (pH 7.5), 0.1% Triton X-100, 10 mM CaCl_2_ and 10 mM MgCl_2_ and then incubated for 24 h at 37°C with gentle agitation in the same buffer as described above [11].

### Quantitative bacteriolytic hydrolase assays

Bacteriolysis of lyophilized *S. aureus* by extracellular hydrolases was quantified as previously described [57]. Equivalent amounts (100 µg) of concentrated supernatants of strain ATCC 25923 were treated with various concentrations of drug or vehicle and were subsequently added to a suspension of autoclaved and lyophilized *S. aureus* ATCC 25923 (1 mg/mL) in 100 mM Tris-HCl (pH 8.0) and incubated at 37°C with shaking. Lytic activity was recorded by measuring the progressive decrease in absorbance (OD_600_).

### Quantification of eDNA in the cell-free supernatants from the biofilms

*S. aureus* ATCC 25923 biofilms were grown in Costar 3614 plates (Corning Life Sciences), and MOL (dissolved in DMSO) was added to eight of the cultures to obtain final concentrations of 1/16 ∼8× MIC, respectively. After 24 h, the plates were chilled at 4°C for 1 h, and 1 µL of 0.5 M EDTA was added to each well. The supernatants were discarded, and unwashed biofilm were harvested by resuspension in 50 mM Tris·HCl/10 mM ETDA/500 mM NaCl, pH 8.0 and transferred into chilled tubes. After centrifugation for 5 min at 4°C and 18,000× *g*, 100 µL of each supernatant was transferred to a tube containing 300 µl of TE buffer (10 mM Tris·HCl/1 mM EDTA, pH 8.0), and extracted once with an equal volume of phenol/chloroform/isoamyl alcohol (25∶24∶1) and once with chloroform/isoamyl alcohol (24∶1). The aqueous phase of each sample was then mixed with 3 vol of ice-cold 100% (vol/vol) ethanol and 1/10 volume of 3 M Na-acetate (pH 5.2) and stored at −20°C. The next day, the ethanol-precipitated DNA was collected by centrifugation for 20 min at 4°C and 18,000× *g*, washed with ice-cold 70% (vol/vol) ethanol, air-dried, and dissolved in 20 µL of TE buffer (9). The concentration and purity of the purified DNA were determined spectrophotometrically by the absorbance ratio A260/A280 using NanoDrop 2000 (Thermo Scientific) [58].

## Supporting Information

Table S1 Genes with expression changes of at least twofold in *S. aureus* ATCC25923 exposed to MOL. *^a^*Genes with expression changes upon treatment with 8 µg/mL magnolol (MOL). *^b^*Mu50 genome ORF number. *^c^*Fold change refers to expression increases or decreases for upregulated or downregulated genes, respectively.
(DOC)

Table S2 Genes involved in autolysis or known as related regulators affected by MOL. *^a^* − indicates reduction and + indicates increase; NS, not significant.
(DOC)

Table S3 The expression of genes involved in biofilm affected by MOL. *^a^* − indicates reduction and + indicates increase; NS, not significant.
(DOC)

Table S4 Primers used in real-time RT-PCR with SYBR green probes. *^a^* ORF, open reading frame.
(DOC)
