# Supplementary material for: Transcriptional and Functional Analysis of the Effects of Magnolol: Inhibition of Autolysis and Biofilms in Staphylococcus aureus
Source: PLoS One. 2011 Oct 28;6(10):e26833. doi: 10.1371/journal.pone.0026833 (PMC3203910; doi:10.1371/journal.pone.0026833)
Supplement: Table S1 — Genes with expression changes of at least twofold in S. aureus ATCC25923 exposed to MOL. aGenes with expression changes upon treatment with 8 µg/mL magnolol (MOL). bMu50 genome ORF number. cFold change refers to expression increases or decreases for upregulated or downregulated genes, respectively. (DOC) [file pone.0026833.s001.doc]

| **ORF no.** *^a^* | **Gene** | **Product or putative function** | **Fold change***^c^* | **Functional category** |
| --- | --- | --- | --- | --- |
| SAS072 |  | hypothetical protein, similar to transposase for IS232 | 2.1 | [Transposon and IS](http://www.bio.nite.go.jp/dogan/GeneSearchResult?GENE_LIST_TYPE=1&type=504&GENOME_LIST=n315G1&CLASS_ID=34.05&WITH_GENE_MAP=1) |
| SAS070 |  | hypothetical protein, similar to transposase for IS232 | 3.1 | [Transposon and IS](http://www.bio.nite.go.jp/dogan/GeneSearchResult?GENE_LIST_TYPE=1&type=504&GENOME_LIST=n315G1&CLASS_ID=34.05&WITH_GENE_MAP=1) |
| SA0137 |  | hypothetical protein, similar to transport system protein | -4.5 | [Transport / binding proteins and lipoproteins](http://www.bio.nite.go.jp/dogan/GeneSearchResult?GENE_LIST_TYPE=1&type=504&GENOME_LIST=n315G1&CLASS_ID=31.02&WITH_GENE_MAP=1) |
| SA2074 | modA | probable molybdate-binding protein | -2.5 | [Transport / binding proteins and lipoproteins](http://www.bio.nite.go.jp/dogan/GeneSearchResult?GENE_LIST_TYPE=1&type=504&GENOME_LIST=n315G1&CLASS_ID=31.02&WITH_GENE_MAP=1) |
| SA0214 | uhpT | hexose phosphate transport protein | -2.5 | [Transport / binding proteins and lipoproteins](http://www.bio.nite.go.jp/dogan/GeneSearchResult?GENE_LIST_TYPE=1&type=504&GENOME_LIST=n315G1&CLASS_ID=31.02&WITH_GENE_MAP=1) |
| SA0168 |  | hypothetical protein, similar to probable permease of ABC transporter | -2.8 | [Transport / binding proteins and lipoproteins](http://www.bio.nite.go.jp/dogan/GeneSearchResult?GENE_LIST_TYPE=1&type=504&GENOME_LIST=n315G1&CLASS_ID=31.02&WITH_GENE_MAP=1) |
| SA1140 | glpF | glycerol uptake facilitator | -2.5 | [Transport / binding proteins and lipoproteins](http://www.bio.nite.go.jp/dogan/GeneSearchResult?GENE_LIST_TYPE=1&type=504&GENOME_LIST=n315G1&CLASS_ID=31.02&WITH_GENE_MAP=1) |
| SA0368 |  | hypothetical protein, similar to proton/sodium-glutamate symport protein | -3.4 | [Transport / binding proteins and lipoproteins](http://www.bio.nite.go.jp/dogan/GeneSearchResult?GENE_LIST_TYPE=1&type=504&GENOME_LIST=n315G1&CLASS_ID=31.02&WITH_GENE_MAP=1) |
| SA1224 |  | ABC transporter (ATP-binding protein) homolog | -3.4 | Transport / binding proteins and lipoproteins |
| SA0207 |  | hypothetical protein, similar to maltose/maltodextrin-binding protein | -3.9 | Transport / binding proteins and lipoproteins |
| SA2072 |  | molybdenum transport ATP-binding protein ModC | -6.4 | Transport / binding proteins and lipoproteins |
| SA2135 |  | hypothetical protein, similar to sodium/glutamate symporter | -9.5 | Transport / binding proteins and lipoproteins |
| SA2302 |  | hypothetical protein, similar to ABC transporter | -11.5 | Transport / binding proteins and lipoproteins |
| SA1987 |  | glycine betaine transporter opuD homolog | 2.2 | Transport / binding proteins and lipoproteins |
| SA2166 |  | hypothetical protein, simialr to cationic transporter | 2.0 | Transport / binding proteins and lipoproteins |
| SA2237 | opuCA | glycine betaine/carnitine/choline ABC transporter (ATP-bindin) opuCA | 2.4 | Transport / binding proteins and lipoproteins |
| SA1505 | lysP | lysine-specific permease | 2.6 | Transport / binding proteins and lipoproteins |
| SA2493 | vraE | hypothetical protein, similar to ABC transporter (permease) | 2.7 | Transport / binding proteins and lipoproteins |
| SA0294 |  | hypothetical protein, similar to branched-chain amino acid uptake carrier | 2.6 | Transport / binding proteins and lipoproteins |
| SA2293 | gntP | gluconate permease | 4.3 | Transport / binding proteins and lipoproteins |
| SA2149 |  | hypothetical protein, simialr to ABC transporter, ATP-binding protein | 48.6 | Transport / binding proteins and lipoproteins |
| SA1042 | pyrP | uracil permease | -2.0 | Transport / binding proteins and lipoproteins |
| SA2250 |  | hypothetical protein, similar to antibiotic resistance protein | -2.1 | Transport / binding proteins and lipoproteins |
| SA1341 |  | hypothetical protein, similar to export protein SpcT protein | -2.3 | Transport / binding proteins and lipoproteins |
| SA1580 |  | multidrug resistance protein homolog | -2.1 | Transport / binding proteins and lipoproteins |
| SA1547 | ptaA | PTS system, N-acetylglucosamine-specific IIABC component | -2.2 | Transport / binding proteins and lipoproteins |
| SA0201 | rlp | RGD-containing lipoprotein | -2.0 | Transport / binding proteins and lipoproteins |
| SA0200 |  | hypothetical protein, similar to dipeptide transporter protein dppC | -2.0 | Transport / binding proteins and lipoproteins |
| SA0217 |  | hypothetical protein, similar to periplasmic-iron-binding protein BitC | -2.2 | Transport / binding proteins and lipoproteins |
| SA1190 | alsT | amino acid carrier protein (sodium/alanine symporter) | -2.3 | Transport / binding proteins and lipoproteins |
| SA2145 | tcaB | TcaB protein | -3.4 | Transport / binding proteins and lipoproteins |
| SA0110 | sirB | lipoprotein | -2.6 | Transport / binding proteins and lipoproteins |
| SA2255 |  | oligopeptide transporter putative substrate binding domain | -2.3 | Transport / binding proteins and lipoproteins |
| SA0295 |  | hypothetical protein, similar to outer membrane protein precursor | -2.3 | Transport / binding proteins and lipoproteins |
| SA2216 |  | hypothetical protein, 3imilar to ABC transporter, ATP-binding protein | -2.5 | Transport / binding proteins and lipoproteins |
| SA1732 |  | hypothetical protein, similar to sodium-dependent transporter | -2.5 | Transport / binding proteins and lipoproteins |
| SA0794 | dltB | DltB membrane protein | -2.6 | Transport / binding proteins and lipoproteins |
| SA2251 |  | oligopeptide transporter putative ATPase domain | -2.6 | Transport / binding proteins and lipoproteins |
| SA2145 | tcaB | TcaB protein | -3.7 | Transport / binding proteins and lipoproteins |
| SA2411 |  | hypothetical protein, similar to magnesium citrate secondary transporter | -3.5 | Transport / binding proteins and lipoproteins |
| SA1270 |  | hypothetical protein, similar to amino acid pearmease | -3.0 | Transport / binding proteins and lipoproteins |
| SA0183 | glcA | PTS enzyme II (EC 2.7.1.69), glucose-specific, factor IIA homologue | -2.6 | Transport / binding proteins and lipoproteins |
| SA0198 | oppF | oligopeptide transport ATP-binding protein | -3.0 | Transport / binding proteins and lipoproteins |
| SA0981 | isdF | hypothetical protein, similar to ferrichrome ABC transporter | -3.3 | Transport / binding proteins and lipoproteins |
| SA1183 | opuD | glycine betaine transporter | -3.4 | Transport / binding proteins and lipoproteins |
| SA0135 |  | hypothetical protein, similar to phosphonates transport permease | -3.1 | Transport / binding proteins and lipoproteins |
| SA1979 |  | hypothetical protein, similar toferrichrome ABC transporter (binding prote) | -3.1 | Transport / binding proteins and lipoproteins |
| SA0479 | nupC | pyrimidine nucleoside transport protein | -3.3 | Transport / binding proteins and lipoproteins |
| SA0980 | isdE | hypothetical protein, similar to ferrichrome ABC transporter | -4.1 | Transport / binding proteins and lipoproteins |
| SA1183 | opuD | glycine betaine transporter | -3.3 | Transport / binding proteins and lipoproteins |
| SA2172 | gltT | proton/sodium-glutamate symport protein | -3.3 | Transport / binding proteins and lipoproteins |
| SA0951 | potB | spermidine/putrescine ABC transporter homolog | -5.2 | Transport / binding proteins and lipoproteins |
| SA0111 | sirA | lipoprotein | -5.1 | Transport / binding proteins and lipoproteins |
| SA0206 | msmX | multiple sugar-binding transport ATP-binding protein | -4.8 | Transport / binding proteins and lipoproteins |
| SA2253 | opp-1C | oligopeptide transporter putative membrane permease domain | -3.5 | Transport / binding proteins and lipoproteins |
| SA0325 | glpT | glycerol-3-phosphate transporter | -5.2 | Transport / binding proteins and lipoproteins |
| SA0566 |  | hypothetical protein, similar to iron-binding protein | -4.8 | Transport / binding proteins and lipoproteins |
| SA0136 |  | hypothetical protein, similar to phosphonates transport permease | -4.4 | Transport / binding proteins and lipoproteins |
| SA1239 | braB | branched-chain amino acid carrier protein | -4.2 | Transport / binding proteins and lipoproteins |
| SA2132 |  | hypothetical protein, 4imilar to ABC transporter (ATP-binding protein) | -4.7 | Transport / binding proteins and lipoproteins |
| SA0796 | dltD | poly(glycerophosphate chain) D-alanine transfer protein | -4.9 | Transport / binding proteins and lipoproteins |
| SA0950 | potA | spermidine/putrescine ABC transporter, ATP-binding protein homolog | -7.6 | Transport / binding proteins and lipoproteins |
| SA0374 | pbuX | xanthine permease | -4.7 | Transport / binding proteins and lipoproteins |
| SA2252 | opp-1D | oligopeptide transporter putative ATPase domain | -4.1 | Transport / binding proteins and lipoproteins |
| SA2254 | opp-1B | oligopeptide transporter putative membrane permease domain | -4.4 | Transport / binding proteins and lipoproteins |
| SA0138 |  | hypothetical protein, similar to alkylphosphonate ABC tranporter | -5.9 | Transport / binding proteins and lipoproteins |
| SA2303 |  | hypothetical protein, imilar to membrane spanning protein | -6.8 | Transport / binding proteins and lipoproteins |
| SA2074 | modA | probable molybdate-binding protein | -12.8 | Transport / binding proteins and lipoproteins |
| SA0952 | potC | spermidine/putrescine ABC transporter homolog | -6.2 | Transport / binding proteins and lipoproteins |
| SA2073 | modB | probable molybdenum transport permease | -5.6 | Transport / binding proteins and lipoproteins |
| SA0325 | glpT | glycerol-3-phosphate transporter | -8.5 | Transport / binding proteins and lipoproteins |
| SA2303 |  | hypothetical protein, 5imilar to membrane spanning protein | -14.33 | Transport / binding proteins and lipoproteins |
| SA0272 |  | hypothetical protein, similar to transmembrane protein Tmp7 | -14.2 | Transport / binding proteins and lipoproteins |
| SA2408 | cudT | choline transporter | -13.4 | Transport / binding proteins and lipoproteins |
| SA0849 |  | hypothetical protein, similar to peptide binding protein OppA | 2.0 | Transport / binding proteins and lipoproteins |
| SA0163 |  | hypothetical protein, similar to cation-efflux system membrane protein CzcD | 2.1 | Transport / binding proteins and lipoproteins |
| SA2081 |  | hypothetical protein, similar to urea transporter | 2.4 | Transport / binding proteins and lipoproteins |
| SA1948 | czrB | cation-efflux system membrane protein homolog | 2.0 | Transport / binding proteins and lipoproteins |
| SA2236 | opuCB | probable glycine betaine/carnitine/choline ABC transporter (membrane p) opuCB | 2.6 | Transport / binding proteins and lipoproteins |
| SA2235 | opuCC | glycine betaine/carnitine/choline ABC transporter (osmoprotec) opuCC | 2.7 | Transport / binding proteins and lipoproteins |
| SA0297 |  | hypothetical protein, similar to ABC transporter ATP-binding protein | 2.6 | Transport / binding proteins and lipoproteins |
| SA2492 | vraD | hypothetical protein, similar to ABC transporter | 3.0 | Transport / binding proteins and lipoproteins |
| SA2234 | opuCD | probable glycine betaine/carnitine/choline ABC transporter (membrane p) opuCD | 2.7 | Transport / binding proteins and lipoproteins |
| SA2396 |  | hypothetical protein, similar to amino acid transporter | 3.8 | Transport / binding proteins and lipoproteins |
| SA0229 |  | hypothetical protein, similar to nickel ABC transporter nickel-binding protein | 4.3 | Transport / binding proteins and lipoproteins |
| SA2339 |  | hypothetical protein, similar to antibiotic transport-associated protein | 37.4 | Transport / binding proteins and lipoproteins |
| SA0706 |  | hypothetical protein, similar to comF operon protein 3 | 2.1 | [Transformation / competence](http://www.bio.nite.go.jp/dogan/GeneSearchResult?GENE_LIST_TYPE=1&type=504&GENOME_LIST=n315G1&CLASS_ID=31.10&WITH_GENE_MAP=1) |
| SA0540 |  | conserved hypothetical protein | -2.0 | similar to unknown proteins |
| SA2277 |  | conserved hypothetical protein | -2.0 | similar to unknown proteins |
| SA0667 |  | conserved hypothetical protein | -2.1 | similar to unknown proteins |
| SA0228 |  | hypothetical protein | -2.0 | similar to unknown proteins |
| SA0477 |  | conserved hypothetical protein | -2.0 | similar to unknown proteins |
| SA1426 |  | conserved hypothetical protein | -2.1 | similar to unknown proteins |
| SA1868 |  | conserved hypothetical protein | -2.1 | similar to unknown proteins |
| SA1576 |  | conserved hypothetical protein | -2.0 | similar to unknown proteins |
| SA0447 |  | conserved hypothetical protein | -2.0 | similar to unknown proteins |
| SA1918 |  | conserved hypothetical protein | -2.1 | similar to unknown proteins |
| SA1928 |  | hypothetical protein | -2.2 | similar to unknown proteins |
| SA1976 |  | conserved 7imilar7r7io protein | -2.1 | similar to unknown proteins |
| SA2106 |  | hypothetical protein, similar to protein of pXO2-46 | -2.5 | similar to unknown proteins |
| SA0165 |  | hypothetical protein, similar to alpha-helical coiled-coil protein SrpF | -2.1 | similar to unknown proteins |
| SA2313 |  | conserved hypothetical protein | -2.6 | similar to unknown proteins |
| SA2106 |  | hypothetical protein, similar to protein of pXO2-46 | -2.3 | similar to unknown proteins |
| SA0975 |  | conserved hypothetical protein | -2.1 | similar to unknown proteins |
| SA1275 |  | conserved hypothetical protein | -2.2 | similar to unknown proteins |
| SA1612 |  | conserved hypothetical protein | -2.4 | similar to unknown proteins |
| SA1240 |  | conserved hypothetical protein | -2.2 | similar to unknown proteins |
| SA0966 |  | conserved hypothetical protein | -2.3 | similar to unknown proteins |
| SA2163 |  | hypothetical protein | -2.4 | similar to unknown proteins |
| SA1867 |  | conserved hypothetical protein | -3.0 | similar to unknown proteins |
| SA0601 |  | conserved hypothetical protein | -2.0 | similar to unknown proteins |
| SA0089 |  | hypothetical protein, similar to DNA helicase | -2.4 | similar to unknown proteins |
| SA2448 |  | conserved hypothetical protein | -2.2 | similar to unknown proteins |
| SA0890 |  | conserved hypothetical protein | -3.1 | similar to unknown proteins |
| SA2329 | cidA | conserved hypothetical protein | -3.3 | similar to unknown proteins |
| SA2131 |  | conserved hypothetical protein | -2.3 | similar to unknown proteins |
| SA0609 |  | conserved hypothetical protein | -2.4 | similar to unknown proteins |
| SA2377 |  | conserved hypothetical protein | -2.2 | similar to unknown proteins |
| SA1578 |  | conserved hypothetical protein | -2.3 | similar to unknown proteins |
| SA1173 |  | conserved hypothetical protein | -2.7 | similar to unknown proteins |
| SA0257 |  | conserved hypothetical protein | -2.5 | similar to unknown proteins |
| SA0840 |  | conserved hypothetical protein | -4.0 | similar to unknown proteins |
| SA1295 |  | conserved hypothetical protein | -2.4 | similar to unknown proteins |
| SA2276 |  | conserved hypothetical protein | -2.7 | similar to unknown proteins |
| SA0979 | isdD | conserved hypothetical protein | -3.2 | similar to unknown proteins |
| SA0478 |  | conserved hypothetical protein | -3.1 | similar to unknown proteins |
| SA1849 |  | conserved hypothetical protein | -3.1 | similar to unknown proteins |
| SA0789 |  | conserved hypothetical protein | -3.2 | similar to unknown proteins |
| SA2133 |  | conserved hypothetical protein | -3.0 | similar to unknown proteins |
| SA1294 |  | conserved hypothetical protein | -2.7 | similar to unknown proteins |
| SA0739 |  | conserved hypothetical protein | -3.8 | similar to unknown proteins |
| SA1723 |  | conserved hypothetical protein | -3.7 | similar to unknown proteins |
| SA1850 |  | conserved hypothetical protein | -2.9 | similar to unknown proteins |
| SA1867 |  | conserved hypothetical protein | -4.3 | similar to unknown proteins |
| SA0517 |  | conserved hypothetical protein | -3.6 | similar to unknown proteins |
| SA0982 | srtB | NPQTN specific sortase B | -4.4 | similar to unknown proteins |
| SA0499 |  | conserved hypothetical protein | -3.1 | similar to unknown proteins |
| SA2133 |  | conserved hypothetical protein | -3.7 | similar to unknown proteins |
| SA1705 |  | conserved hypothetical protein | -3.6 | similar to unknown proteins |
| SA0949 |  | conserved hypothetical protein | -4.1 | similar to unknown proteins |
| SA0213 |  | conserved hypothetical protein | -12.0 | similar to unknown proteins |
| SA2332 |  | hypothetical protein, similar to secretory antigen precursor SsaA | -4.8 | similar to unknown proteins |
| SA1265 |  | conserved hypothetical protein | -6.1 | similar to unknown proteins |
| SA0308 |  | conserved hypothetical protein | -4.5 | similar to unknown proteins |
| SA0266 |  | conserved hypothetical protein | -3.9 | similar to unknown proteins |
| SA0983 | isdG | conserved hypothetical protein | -4.4 | similar to unknown proteins |
| SA0308 |  | conserved hypothetical protein | -4.8 | similar to unknown proteins |
| SA2407 |  | conserved hypothetical protein | -4.4 | similar to unknown proteins |
| SA0274 |  | conserved hypothetical protein | -9.1 | similar to unknown proteins |
| SA0976 | isdB | conserved hypothetical protein | -9.2 | similar to unknown proteins |
| SA0518 |  | conserved hypothetical protein | -4.5 | similar to unknown proteins |
| SA0275 |  | conserved hypothetical protein | -9.4 | similar to unknown proteins |
| SA0269 |  | hypothetical protein | -10.1 | similar to unknown proteins |
| SA0753 |  | conserved hypothetical protein | 2.0 | similar to unknown proteins |
| SA1980 |  | conserved hypothetical protein | 2.0 | similar to unknown proteins |
| SA0725 |  | conserved hypothetical protein | 2.1 | similar to unknown proteins |
| SA1990 |  | conserved hypothetical protein | 2.0 | similar to unknown proteins |
| SA1657 |  | conserved hypothetical protein | 2.2 | similar to unknown proteins |
| SA1327 |  | conserved hypothetical protein | 2.1 | similar to unknown proteins |
| SA0721 |  | conserved hypothetical protein | 2.0 | similar to unknown proteins |
| SA0360 |  | conserved hypothetical protein | 2.4 | similar to unknown proteins |
| SA1453 |  | conserved hypothetical protein | 2.0 | similar to unknown proteins |
| SA2298 |  | conserved hypothetical protein | 2.1 | similar to unknown proteins |
| SA1702 |  | conserved hypothetical protein | 2.1 | similar to unknown proteins |
| SA0860 |  | conserved hypothetical protein | 2.2 | similar to unknown proteins |
| SA1532 |  | conserved hypothetical protein | 2.3 | similar to unknown proteins |
| SA0621 |  | conserved hypothetical protein | 2.2 | similar to unknown proteins |
| SA0323 | mepA | conserved hypothetical protein | 2.2 | similar to unknown proteins |
| SA1293 |  | conserved hypothetical protein | 2.3 | similar to unknown proteins |
| SA1544 |  | hypothetical protein, similar to soluble hydrogenase 42 kD subunit | 2.2 | similar to unknown proteins |
| SA2259 |  | conserved hypothetical protein | 2.0 | similar to unknown proteins |
| SA1981 |  | conserved hypothetical protein | 2.1 | similar to unknown proteins |
| SA0296 |  | conserved hypothetical protein | 2.2 | similar to unknown proteins |
| SA0860 |  | conserved hypothetical protein | 2.3 | similar to unknown proteins |
| SA0230 |  | conserved hypothetical protein | 3.3 | similar to unknown proteins |
| SA2328 | cidB | conserved hypothetical protein | 2.3 | similar to unknown proteins |
| SA0862 |  | conserved hypothetical protein | 2.3 | similar to unknown proteins |
| SA0380 |  | conserved hypothetical protein [Pathogenicity island SaPIn2] | 2.7 | similar to unknown proteins |
| SA1946 |  | conserved hypothetical protein | 2.2 | similar to unknown proteins |
| SA0861 |  | conserved hypothetical protein | 2.5 | similar to unknown proteins |
| SA2365 |  | hypothetical protein, similar to short chain oxidoreductase | 2.7 | similar to unknown proteins |
| SA2439 |  | conserved hypothetical protein | 2.7 | similar to unknown proteins |
| SA0612 |  | conserved hypothetical protein | 2.7 | similar to unknown proteins |
| SA1543 |  | conserved hypothetical protein | 2.6 | similar to unknown proteins |
| SA0722 |  | conserved hypothetical protein | 2.4 | similar to unknown proteins |
| SA0413 |  | conserved hypothetical protein | 3.0 | similar to unknown proteins |
| SA0800 |  | conserved hypothetical protein | 3.2 | similar to unknown proteins |
| SA2440 |  | hypothetical protein | 2.9 | similar to unknown proteins |
| SA2374 |  | conserved hypothetical protein | 2.4 | similar to unknown proteins |
| SA0085 |  | conserved hypothetical protein | 2.7 | similar to unknown proteins |
| SA2491 |  | conserved hypothetical protein | 4.2 | similar to unknown proteins |
| SA0079 |  | conserved hypothetical protein | 2.7 | similar to unknown proteins |
| SA2325 |  | conserved hypothetical protein | 3.1 | similar to unknown proteins |
| SA1686 |  | conserved hypothetical protein | 3.0 | similar to unknown proteins |
| SA2262 |  | conserved hypothetical protein | 3.4 | similar to unknown proteins |
| SA0703 |  | conserved hypothetical protein | 3.7 | similar to unknown proteins |
| SA2403 |  | conserved hypothetical protein | 3.6 | similar to unknown proteins |
| SA1057 |  | conserved hypothetical protein | 4.0 | similar to unknown proteins |
| SA1235 |  | conserved hypothetical protein | 4.2 | similar to unknown proteins |
| SA0481 |  | conserved hypothetical protein | 3.7 | similar to unknown proteins |
| SA0412 |  | conserved hypothetical protein | 4.8 | similar to unknown proteins |
| SA0381 |  | conserved hypothetical protein [Pathogenicity island SaPIn2] | 4.0 | similar to unknown proteins |
| SA2380 |  | conserved hypothetical protein | 4.5 | similar to unknown proteins |
| SA2150 |  | hypothetical protein | 101.3 | similar to unknown proteins |
| SA1515 | phoR | alkaline phosphatase synthesis sensor protein | 2.2 | [Sensors (signal transduction)](http://www.bio.nite.go.jp/dogan/GeneSearchResult?GENE_LIST_TYPE=1&type=504&GENOME_LIST=n315G1&CLASS_ID=31.03&WITH_GENE_MAP=1) |
| SA1149 | glnR | glutamine synthetase repressor | -2.0 | [RNA synthesis](http://www.bio.nite.go.jp/dogan/GeneSearchResult?GENE_LIST_TYPE=1&type=504&GENOME_LIST=n315G1&CLASS_ID=33.05&WITH_GENE_MAP=1) |
| SA2115 |  | hypothetical protein, similar to transcriptional regulator | -2.2 | [RNA synthesis](http://www.bio.nite.go.jp/dogan/GeneSearchResult?GENE_LIST_TYPE=1&type=504&GENOME_LIST=n315G1&CLASS_ID=33.05&WITH_GENE_MAP=1) |
| SA1676 |  | hypothetical protein, similar to regulatory protein (pfoS/R) | -2.0 | [RNA synthesis](http://www.bio.nite.go.jp/dogan/GeneSearchResult?GENE_LIST_TYPE=1&type=504&GENOME_LIST=n315G1&CLASS_ID=33.05&WITH_GENE_MAP=1) |
| SA0476 |  | hypothetical protein, similar to transcription regulator GntR family | -2.1 | [RNA synthesis](http://www.bio.nite.go.jp/dogan/GeneSearchResult?GENE_LIST_TYPE=1&type=504&GENOME_LIST=n315G1&CLASS_ID=33.05&WITH_GENE_MAP=1) |
| SA1872 | rsbU | sigmaB regulation protein RsbU | -2.3 | [RNA synthesis](http://www.bio.nite.go.jp/dogan/GeneSearchResult?GENE_LIST_TYPE=1&type=504&GENOME_LIST=n315G1&CLASS_ID=33.05&WITH_GENE_MAP=1) |
| SA2092 |  | hypothetical protein, similar to transcription regulator | -2.8 | [RNA synthesis](http://www.bio.nite.go.jp/dogan/GeneSearchResult?GENE_LIST_TYPE=1&type=504&GENOME_LIST=n315G1&CLASS_ID=33.05&WITH_GENE_MAP=1) |
| SA2320 |  | hypothetical protein, similar to regulatory protein pfoR | -2.6 | [RNA synthesis](http://www.bio.nite.go.jp/dogan/GeneSearchResult?GENE_LIST_TYPE=1&type=504&GENOME_LIST=n315G1&CLASS_ID=33.05&WITH_GENE_MAP=1) |
| SA0251 | lytR | two-component response regulator | -2.7 | [RNA synthesis](http://www.bio.nite.go.jp/dogan/GeneSearchResult?GENE_LIST_TYPE=1&type=504&GENOME_LIST=n315G1&CLASS_ID=33.05&WITH_GENE_MAP=1) |
| SA2060 |  | hypothetical protein, similar to transcription regulator MarR family | -3.8 | [RNA synthesis](http://www.bio.nite.go.jp/dogan/GeneSearchResult?GENE_LIST_TYPE=1&type=504&GENOME_LIST=n315G1&CLASS_ID=33.05&WITH_GENE_MAP=1) |
| SA1897 |  | hypothetical protein, 12imilar to 12imilar12r12ional activator TenA | -4.5 | [RNA synthesis](http://www.bio.nite.go.jp/dogan/GeneSearchResult?GENE_LIST_TYPE=1&type=504&GENOME_LIST=n315G1&CLASS_ID=33.05&WITH_GENE_MAP=1) |
| SA1041 | pyrR | pyrimidine operon repressor chainA | -6.0 | [RNA synthesis](http://www.bio.nite.go.jp/dogan/GeneSearchResult?GENE_LIST_TYPE=1&type=504&GENOME_LIST=n315G1&CLASS_ID=33.05&WITH_GENE_MAP=1) |
| SA1174 | lexA | SOS regulatory LexA protein | 2.2 | [RNA synthesis](http://www.bio.nite.go.jp/dogan/GeneSearchResult?GENE_LIST_TYPE=1&type=504&GENOME_LIST=n315G1&CLASS_ID=33.05&WITH_GENE_MAP=1) |
| SA1844 | agrA | accessory gene regulator A | 2.3 | [RNA synthesis](http://www.bio.nite.go.jp/dogan/GeneSearchResult?GENE_LIST_TYPE=1&type=504&GENOME_LIST=n315G1&CLASS_ID=33.05&WITH_GENE_MAP=1) |
| SA1516 | phoP | alkaline phosphatase synthesis transcriptional regulatory protein | 2.1 | [RNA synthesis](http://www.bio.nite.go.jp/dogan/GeneSearchResult?GENE_LIST_TYPE=1&type=504&GENOME_LIST=n315G1&CLASS_ID=33.05&WITH_GENE_MAP=1) |
| SA1248 |  | truncated (putative response regulator ArlR) | 2.0 | [RNA synthesis](http://www.bio.nite.go.jp/dogan/GeneSearchResult?GENE_LIST_TYPE=1&type=504&GENOME_LIST=n315G1&CLASS_ID=33.05&WITH_GENE_MAP=1) |
| SA2357 |  | hypothetical protein, similar to regulatory protein (pfoS/R) | 2.3 | [RNA synthesis](http://www.bio.nite.go.jp/dogan/GeneSearchResult?GENE_LIST_TYPE=1&type=504&GENOME_LIST=n315G1&CLASS_ID=33.05&WITH_GENE_MAP=1) |
| SA2364 |  | hypothetical protein, similar to transcription regulator acrR | 3.4 | [RNA synthesis](http://www.bio.nite.go.jp/dogan/GeneSearchResult?GENE_LIST_TYPE=1&type=504&GENOME_LIST=n315G1&CLASS_ID=33.05&WITH_GENE_MAP=1) |
| SA2296 |  | hypothetical protein, 12imilar to transcriptional regulator, MerR family | 2.5 | [RNA synthesis](http://www.bio.nite.go.jp/dogan/GeneSearchResult?GENE_LIST_TYPE=1&type=504&GENOME_LIST=n315G1&CLASS_ID=33.05&WITH_GENE_MAP=1) |
| SA2108 |  | hypothetical protein, 12imilar to transcription regulator, RpiR family | 2.4 | [RNA synthesis](http://www.bio.nite.go.jp/dogan/GeneSearchResult?GENE_LIST_TYPE=1&type=504&GENOME_LIST=n315G1&CLASS_ID=33.05&WITH_GENE_MAP=1) |
| SA2002 |  | hypothetical protein, similar to transcription regulator MerR family | 3.0 | [RNA synthesis](http://www.bio.nite.go.jp/dogan/GeneSearchResult?GENE_LIST_TYPE=1&type=504&GENOME_LIST=n315G1&CLASS_ID=33.05&WITH_GENE_MAP=1) |
| SA2340 |  | hypothetical protein, similar to transcriptional regulator tetR-family | 2.4 | [RNA synthesis](http://www.bio.nite.go.jp/dogan/GeneSearchResult?GENE_LIST_TYPE=1&type=504&GENOME_LIST=n315G1&CLASS_ID=33.05&WITH_GENE_MAP=1) |
| SA1411 | hrcA | Heat-inducible transcriptional repressor | 2.5 | [RNA synthesis](http://www.bio.nite.go.jp/dogan/GeneSearchResult?GENE_LIST_TYPE=1&type=504&GENOME_LIST=n315G1&CLASS_ID=33.05&WITH_GENE_MAP=1) |
| SA0573 | sarA | staphylococcal accessory regulator A | 3.1 | [RNA synthesis](http://www.bio.nite.go.jp/dogan/GeneSearchResult?GENE_LIST_TYPE=1&type=504&GENOME_LIST=n315G1&CLASS_ID=33.05&WITH_GENE_MAP=1) |
| SA0261 |  | hypothetical protein, similar to rbs operon repressor RbsR | 3.4 | [RNA synthesis](http://www.bio.nite.go.jp/dogan/GeneSearchResult?GENE_LIST_TYPE=1&type=504&GENOME_LIST=n315G1&CLASS_ID=33.05&WITH_GENE_MAP=1) |
| SA2458 | icaR | ica operon transcriptional regulator IcaR | 3.6 | [RNA synthesis](http://www.bio.nite.go.jp/dogan/GeneSearchResult?GENE_LIST_TYPE=1&type=504&GENOME_LIST=n315G1&CLASS_ID=33.05&WITH_GENE_MAP=1) |
| SA1947 | czrA | repressor protein | 3.7 | [RNA synthesis](http://www.bio.nite.go.jp/dogan/GeneSearchResult?GENE_LIST_TYPE=1&type=504&GENOME_LIST=n315G1&CLASS_ID=33.05&WITH_GENE_MAP=1) |
| SA0480 | ctsR | transcription repressor of class III stress genes homologue | 4.0 | [RNA synthesis](http://www.bio.nite.go.jp/dogan/GeneSearchResult?GENE_LIST_TYPE=1&type=504&GENOME_LIST=n315G1&CLASS_ID=33.05&WITH_GENE_MAP=1) |
| SA2295 | gntR | gluconate operon transcriptional repressor | 8.4 | [RNA synthesis](http://www.bio.nite.go.jp/dogan/GeneSearchResult?GENE_LIST_TYPE=1&type=504&GENOME_LIST=n315G1&CLASS_ID=33.05&WITH_GENE_MAP=1) |
| SA1700 | vraR | Two-component response regulator | 2.0 | RNA synthesis |
| SA1387 |  | hypothetical protein, similar to ATP-dependent RNA helicase | -2.0 | [RNA modification](http://www.bio.nite.go.jp/dogan/GeneSearchResult?GENE_LIST_TYPE=1&type=504&GENOME_LIST=n315G1&CLASS_ID=33.06&WITH_GENE_MAP=1) |
| SA2502 | rnpA | ribonuclease P protein component | -2.4 | [RNA modification](http://www.bio.nite.go.jp/dogan/GeneSearchResult?GENE_LIST_TYPE=1&type=504&GENOME_LIST=n315G1&CLASS_ID=33.06&WITH_GENE_MAP=1) |
| SA1082 | rimM | probable 16S rRNA processing protein | -4.8 | [RNA modification](http://www.bio.nite.go.jp/dogan/GeneSearchResult?GENE_LIST_TYPE=1&type=504&GENOME_LIST=n315G1&CLASS_ID=33.06&WITH_GENE_MAP=1) |
| SA1083 | trmD | tRNA (guanine-N1)-mehtyltransferase | -4.7 | [RNA modification](http://www.bio.nite.go.jp/dogan/GeneSearchResult?GENE_LIST_TYPE=1&type=504&GENOME_LIST=n315G1&CLASS_ID=33.06&WITH_GENE_MAP=1) |
| SAS093 | rpmH | 50S ribosomal protein L34 | -2.1 | [Protein synthesis](http://www.bio.nite.go.jp/dogan/GeneSearchResult?GENE_LIST_TYPE=1&type=504&GENOME_LIST=n315G1&CLASS_ID=33.07&WITH_GENE_MAP=1) |
| SA0959 |  | GTP-binding elongation factor homolog | -2.1 | [Protein synthesis](http://www.bio.nite.go.jp/dogan/GeneSearchResult?GENE_LIST_TYPE=1&type=504&GENOME_LIST=n315G1&CLASS_ID=33.07&WITH_GENE_MAP=1) |
| SA1102 | frr | ribosome recycling factor | -2.1 | [Protein synthesis](http://www.bio.nite.go.jp/dogan/GeneSearchResult?GENE_LIST_TYPE=1&type=504&GENOME_LIST=n315G1&CLASS_ID=33.07&WITH_GENE_MAP=1) |
| SA1414 | rpsT | 30S ribosomal protein S20 (BS20) | -2.4 | [Protein synthesis](http://www.bio.nite.go.jp/dogan/GeneSearchResult?GENE_LIST_TYPE=1&type=504&GENOME_LIST=n315G1&CLASS_ID=33.07&WITH_GENE_MAP=1) |
| SA0855 | trpS | tryptophanyl-tRNA synthetase | -2.4 | [Protein synthesis](http://www.bio.nite.go.jp/dogan/GeneSearchResult?GENE_LIST_TYPE=1&type=504&GENOME_LIST=n315G1&CLASS_ID=33.07&WITH_GENE_MAP=1) |
| SA0497 | rplJ | 50S ribosomal protein L10 (BL5) | -2.3 | [Protein synthesis](http://www.bio.nite.go.jp/dogan/GeneSearchResult?GENE_LIST_TYPE=1&type=504&GENOME_LIST=n315G1&CLASS_ID=33.07&WITH_GENE_MAP=1) |
| SA1036 | ileS | Ile-tRNA synthetase | -2.0 | [Protein synthesis](http://www.bio.nite.go.jp/dogan/GeneSearchResult?GENE_LIST_TYPE=1&type=504&GENOME_LIST=n315G1&CLASS_ID=33.07&WITH_GENE_MAP=1) |
| SA1716 |  | glutamyl-tRNAGln amidotransferase subunit A | -2.4 | [Protein synthesis](http://www.bio.nite.go.jp/dogan/GeneSearchResult?GENE_LIST_TYPE=1&type=504&GENOME_LIST=n315G1&CLASS_ID=33.07&WITH_GENE_MAP=1) |
| SA1457 | hisS | histidyl-tRNA synthetase | -2.4 | [Protein synthesis](http://www.bio.nite.go.jp/dogan/GeneSearchResult?GENE_LIST_TYPE=1&type=504&GENOME_LIST=n315G1&CLASS_ID=33.07&WITH_GENE_MAP=1) |
| SA1456 | aspS | aspartyl-tRNA synthetase | -2.2 | [Protein synthesis](http://www.bio.nite.go.jp/dogan/GeneSearchResult?GENE_LIST_TYPE=1&type=504&GENOME_LIST=n315G1&CLASS_ID=33.07&WITH_GENE_MAP=1) |
| SA1099 | rpsB | 30S ribosomal protein S2 | -2.4 | [Protein synthesis](http://www.bio.nite.go.jp/dogan/GeneSearchResult?GENE_LIST_TYPE=1&type=504&GENOME_LIST=n315G1&CLASS_ID=33.07&WITH_GENE_MAP=1) |
| SA0496 | rplA | 50S ribosomal protein L1 (BL1) | -2.3 | [Protein synthesis](http://www.bio.nite.go.jp/dogan/GeneSearchResult?GENE_LIST_TYPE=1&type=504&GENOME_LIST=n315G1&CLASS_ID=33.07&WITH_GENE_MAP=1) |
| SA1504 | infC | translation initiation factor IF-3 infC | -2.9 | [Protein synthesis](http://www.bio.nite.go.jp/dogan/GeneSearchResult?GENE_LIST_TYPE=1&type=504&GENOME_LIST=n315G1&CLASS_ID=33.07&WITH_GENE_MAP=1) |
| SA1550 | tyrS | tyrosyl-tRNA synthetase | -2.5 | [Protein synthesis](http://www.bio.nite.go.jp/dogan/GeneSearchResult?GENE_LIST_TYPE=1&type=504&GENOME_LIST=n315G1&CLASS_ID=33.07&WITH_GENE_MAP=1) |
| SA1717 |  | glutamyl-tRNAGln amidotransferase subunit C | -2.9 | [Protein synthesis](http://www.bio.nite.go.jp/dogan/GeneSearchResult?GENE_LIST_TYPE=1&type=504&GENOME_LIST=n315G1&CLASS_ID=33.07&WITH_GENE_MAP=1) |
| SA0986 | pheT | Phe-tRNA synthetase beta chain | -3.0 | [Protein synthesis](http://www.bio.nite.go.jp/dogan/GeneSearchResult?GENE_LIST_TYPE=1&type=504&GENOME_LIST=n315G1&CLASS_ID=33.07&WITH_GENE_MAP=1) |
| SA0486 | gltX | glutamyl-tRNA synthetase | -2.7 | [Protein synthesis](http://www.bio.nite.go.jp/dogan/GeneSearchResult?GENE_LIST_TYPE=1&type=504&GENOME_LIST=n315G1&CLASS_ID=33.07&WITH_GENE_MAP=1) |
| SA0985 | pheS | Phe-tRNA synthetase alpha chain | -2.6 | [Protein synthesis](http://www.bio.nite.go.jp/dogan/GeneSearchResult?GENE_LIST_TYPE=1&type=504&GENOME_LIST=n315G1&CLASS_ID=33.07&WITH_GENE_MAP=1) |
| SA1920 | prfA | peptide chain release factor 1 | -2.8 | [Protein synthesis](http://www.bio.nite.go.jp/dogan/GeneSearchResult?GENE_LIST_TYPE=1&type=504&GENOME_LIST=n315G1&CLASS_ID=33.07&WITH_GENE_MAP=1) |
| SA1579 | leuS | leucyl-tRNA synthetase | -3.0 | [Protein synthesis](http://www.bio.nite.go.jp/dogan/GeneSearchResult?GENE_LIST_TYPE=1&type=504&GENOME_LIST=n315G1&CLASS_ID=33.07&WITH_GENE_MAP=1) |
| SAS052 | rpsD | 30S ribosomal protein S4 | -3.6 | [Protein synthesis](http://www.bio.nite.go.jp/dogan/GeneSearchResult?GENE_LIST_TYPE=1&type=504&GENOME_LIST=n315G1&CLASS_ID=33.07&WITH_GENE_MAP=1) |
| SA1503 | rpmI | 50S ribosomal protein L35 | -3.1 | [Protein synthesis](http://www.bio.nite.go.jp/dogan/GeneSearchResult?GENE_LIST_TYPE=1&type=504&GENOME_LIST=n315G1&CLASS_ID=33.07&WITH_GENE_MAP=1) |
| SA1081 | rpsP | 30S ribosomal protein S16 | -3.4 | [Protein synthesis](http://www.bio.nite.go.jp/dogan/GeneSearchResult?GENE_LIST_TYPE=1&type=504&GENOME_LIST=n315G1&CLASS_ID=33.07&WITH_GENE_MAP=1) |
| SA1502 | rplT | 50S ribosomal protein L20 | -4.6 | [Protein synthesis](http://www.bio.nite.go.jp/dogan/GeneSearchResult?GENE_LIST_TYPE=1&type=504&GENOME_LIST=n315G1&CLASS_ID=33.07&WITH_GENE_MAP=1) |
| SA0354 | rpsR | 30S ribosomal protein S18 | -4.2 | [Protein synthesis](http://www.bio.nite.go.jp/dogan/GeneSearchResult?GENE_LIST_TYPE=1&type=504&GENOME_LIST=n315G1&CLASS_ID=33.07&WITH_GENE_MAP=1) |
| SA1503 | rpmI | 50S ribosomal protein L35 | -3.8 | [Protein synthesis](http://www.bio.nite.go.jp/dogan/GeneSearchResult?GENE_LIST_TYPE=1&type=504&GENOME_LIST=n315G1&CLASS_ID=33.07&WITH_GENE_MAP=1) |
| SA0498 | rplL | 50S ribosomal protein L7/L12 | -4.6 | [Protein synthesis](http://www.bio.nite.go.jp/dogan/GeneSearchResult?GENE_LIST_TYPE=1&type=504&GENOME_LIST=n315G1&CLASS_ID=33.07&WITH_GENE_MAP=1) |
| SA0455 |  | translation initiation inhibitor homologue | 2.1 | [Protein synthesis](http://www.bio.nite.go.jp/dogan/GeneSearchResult?GENE_LIST_TYPE=1&type=504&GENOME_LIST=n315G1&CLASS_ID=33.07&WITH_GENE_MAP=1) |
| SAS042 | rpmG | 50S ribosomal protein L33 | 3.3 | [Protein synthesis](http://www.bio.nite.go.jp/dogan/GeneSearchResult?GENE_LIST_TYPE=1&type=504&GENOME_LIST=n315G1&CLASS_ID=33.07&WITH_GENE_MAP=1) |
| SA2442 |  | preprotein translocase secA homolog | 2.4 | [Protein secretion](http://www.bio.nite.go.jp/dogan/GeneSearchResult?GENE_LIST_TYPE=1&type=504&GENOME_LIST=n315G1&CLASS_ID=31.06&WITH_GENE_MAP=1) |
| SA1360 |  | Xaa-Pro dipeptidase | -7.0 | [Protein modification](http://www.bio.nite.go.jp/dogan/GeneSearchResult?GENE_LIST_TYPE=1&type=504&GENOME_LIST=n315G1&CLASS_ID=33.08&WITH_GENE_MAP=1) |
| SA1855 |  | hypothetical protein, similar to ribosomal-protein-alanine N-acetyltransfer | -2.2 | [Protein modification](http://www.bio.nite.go.jp/dogan/GeneSearchResult?GENE_LIST_TYPE=1&type=504&GENOME_LIST=n315G1&CLASS_ID=33.08&WITH_GENE_MAP=1) |
| SA1063 |  | protein kinase | -2.2 | [Protein modification](http://www.bio.nite.go.jp/dogan/GeneSearchResult?GENE_LIST_TYPE=1&type=504&GENOME_LIST=n315G1&CLASS_ID=33.08&WITH_GENE_MAP=1) |
| SA0884 |  | lipoate-protein ligase homolog | -2.1 | [Protein modification](http://www.bio.nite.go.jp/dogan/GeneSearchResult?GENE_LIST_TYPE=1&type=504&GENOME_LIST=n315G1&CLASS_ID=33.08&WITH_GENE_MAP=1) |
| SA1360 |  | Xaa-Pro dipeptidase | -4.3 | [Protein modification](http://www.bio.nite.go.jp/dogan/GeneSearchResult?GENE_LIST_TYPE=1&type=504&GENOME_LIST=n315G1&CLASS_ID=33.08&WITH_GENE_MAP=1) |
| SA1725 | scpA | Staphopain, Cysteine Proteinase | -9.2 | [Protein modification](http://www.bio.nite.go.jp/dogan/GeneSearchResult?GENE_LIST_TYPE=1&type=504&GENOME_LIST=n315G1&CLASS_ID=33.08&WITH_GENE_MAP=1) |
| SA1409 | dnaK | DnaK protein (HSP70) | 2.4 | [Protein folding](http://www.bio.nite.go.jp/dogan/GeneSearchResult?GENE_LIST_TYPE=1&type=504&GENOME_LIST=n315G1&CLASS_ID=33.09&WITH_GENE_MAP=1) |
| SA1783 |  | hypothetical protein [Bacteriophage phiN315] | -2.2 | [Phage-related functions](http://www.bio.nite.go.jp/dogan/GeneSearchResult?GENE_LIST_TYPE=1&type=504&GENOME_LIST=n315G1&CLASS_ID=34.04&WITH_GENE_MAP=1) |
| SA1765 |  | hypothetical protein [Bacteriophage phiN315] | -2.1 | [Phage-related functions](http://www.bio.nite.go.jp/dogan/GeneSearchResult?GENE_LIST_TYPE=1&type=504&GENOME_LIST=n315G1&CLASS_ID=34.04&WITH_GENE_MAP=1) |
| SA1835 | int | hypothetical protein, 14imilar to integrase [Pathogenicity island SaPIn1] | -2.3 | [Phage-related functions](http://www.bio.nite.go.jp/dogan/GeneSearchResult?GENE_LIST_TYPE=1&type=504&GENOME_LIST=n315G1&CLASS_ID=34.04&WITH_GENE_MAP=1) |
| SA1762 |  | hypothetical protein [Bacteriophage phiN315] | -3.2 | [Phage-related functions](http://www.bio.nite.go.jp/dogan/GeneSearchResult?GENE_LIST_TYPE=1&type=504&GENOME_LIST=n315G1&CLASS_ID=34.04&WITH_GENE_MAP=1) |
| SA0754 |  | hypothetical protein, similar to lactococcal prophage ps3 protein 05 | 2.2 | [Phage-related functions](http://www.bio.nite.go.jp/dogan/GeneSearchResult?GENE_LIST_TYPE=1&type=504&GENOME_LIST=n315G1&CLASS_ID=34.04&WITH_GENE_MAP=1) |
| SA1820 |  | hypothetical protein, similar to bacteriophage terminase small subunit [Pathogenicity island SaPIn1] | 2.3 | [Phage-related functions](http://www.bio.nite.go.jp/dogan/GeneSearchResult?GENE_LIST_TYPE=1&type=504&GENOME_LIST=n315G1&CLASS_ID=34.04&WITH_GENE_MAP=1) |
| SA1095 | xerC | site-specific recombinase XerC homolog | 2.4 | [Phage-related functions](http://www.bio.nite.go.jp/dogan/GeneSearchResult?GENE_LIST_TYPE=1&type=504&GENOME_LIST=n315G1&CLASS_ID=34.04&WITH_GENE_MAP=1) |
| SA0253 | lrgB | antiholin-like protein LrgB | 30.9 | [Phage-related functions](http://www.bio.nite.go.jp/dogan/GeneSearchResult?GENE_LIST_TYPE=1&type=504&GENOME_LIST=n315G1&CLASS_ID=34.04&WITH_GENE_MAP=1) |
| SA2164 |  | hypothetical protein, similar to phage infection protein precursor | 20.5 | [Phage-related functions](http://www.bio.nite.go.jp/dogan/GeneSearchResult?GENE_LIST_TYPE=1&type=504&GENOME_LIST=n315G1&CLASS_ID=34.04&WITH_GENE_MAP=1) |
| SA0252 | lrgA | murein hydrolase regulator LrgA | 35.0 | [Phage-related functions](http://www.bio.nite.go.jp/dogan/GeneSearchResult?GENE_LIST_TYPE=1&type=504&GENOME_LIST=n315G1&CLASS_ID=34.04&WITH_GENE_MAP=1) |
| SA1429 |  | enterotoxin homolog | -2.1 | [Pathogenic factors (toxins and colonization factors)](http://www.bio.nite.go.jp/dogan/GeneSearchResult?GENE_LIST_TYPE=1&type=504&GENOME_LIST=n315G1&CLASS_ID=34.06&WITH_GENE_MAP=1) |
| SA0610 |  | hypothetical protein, similar to lipase LipA | -2.1 | [Pathogenic factors (toxins and colonization factors)](http://www.bio.nite.go.jp/dogan/GeneSearchResult?GENE_LIST_TYPE=1&type=504&GENOME_LIST=n315G1&CLASS_ID=34.06&WITH_GENE_MAP=1) |
| SA1645 | yent1 | enterotoxin Yent1 [Pathogenicity island SaPIn3] | -2.1 | [Pathogenic factors (toxins and colonization factors)](http://www.bio.nite.go.jp/dogan/GeneSearchResult?GENE_LIST_TYPE=1&type=504&GENOME_LIST=n315G1&CLASS_ID=34.06&WITH_GENE_MAP=1) |
| SA1752 | truncated(hlb) | truncated beta-hemolysin | -2.6 | [Pathogenic factors (toxins and colonization factors)](http://www.bio.nite.go.jp/dogan/GeneSearchResult?GENE_LIST_TYPE=1&type=504&GENOME_LIST=n315G1&CLASS_ID=34.06&WITH_GENE_MAP=1) |
| SA1647 | sem | enterotoxin SEM [Pathogenicity island SaPIn3] | -3.6 | [Pathogenic factors (toxins and colonization factors)](http://www.bio.nite.go.jp/dogan/GeneSearchResult?GENE_LIST_TYPE=1&type=504&GENOME_LIST=n315G1&CLASS_ID=34.06&WITH_GENE_MAP=1) |
| SA1646 | sei | extracellular enterotoxin type I precursor [Pathogenicity island SaPIn3] | -2.5 | [Pathogenic factors (toxins and colonization factors)](http://www.bio.nite.go.jp/dogan/GeneSearchResult?GENE_LIST_TYPE=1&type=504&GENOME_LIST=n315G1&CLASS_ID=34.06&WITH_GENE_MAP=1) |
| SA2423 | clfB | Clumping factor B | -2.5 | [Pathogenic factors (toxins and colonization factors)](http://www.bio.nite.go.jp/dogan/GeneSearchResult?GENE_LIST_TYPE=1&type=504&GENOME_LIST=n315G1&CLASS_ID=34.06&WITH_GENE_MAP=1) |
| SA0521 | sdrE | Ser-Asp rich fibrinogen-binding, bone sialoprotein-binding protein | -2.5 | [Pathogenic factors (toxins and colonization factors)](http://www.bio.nite.go.jp/dogan/GeneSearchResult?GENE_LIST_TYPE=1&type=504&GENOME_LIST=n315G1&CLASS_ID=34.06&WITH_GENE_MAP=1) |
| SA0519 | sdrC | Ser-Asp rich fibrinogen-binding, bone sialoprotein-binding protein | -2.5 | [Pathogenic factors (toxins and colonization factors)](http://www.bio.nite.go.jp/dogan/GeneSearchResult?GENE_LIST_TYPE=1&type=504&GENOME_LIST=n315G1&CLASS_ID=34.06&WITH_GENE_MAP=1) |
| SA0520 | sdrD | Ser-Asp rich fibrinogen-binding, bone sialoprotein-binding protein | -2.5 | [Pathogenic factors (toxins and colonization factors)](http://www.bio.nite.go.jp/dogan/GeneSearchResult?GENE_LIST_TYPE=1&type=504&GENOME_LIST=n315G1&CLASS_ID=34.06&WITH_GENE_MAP=1) |
| SA0270 |  | hypothetical protein, similar to secretory antigen precursor SsaA | -2.1 | [Pathogenic factors (toxins and colonization factors)](http://www.bio.nite.go.jp/dogan/GeneSearchResult?GENE_LIST_TYPE=1&type=504&GENOME_LIST=n315G1&CLASS_ID=34.06&WITH_GENE_MAP=1) |
| SA0091 | plc | 1-phosphatidylinositol phosphodiesterase 15imilar15r | -3.0 | [Pathogenic factors (toxins and colonization factors)](http://www.bio.nite.go.jp/dogan/GeneSearchResult?GENE_LIST_TYPE=1&type=504&GENOME_LIST=n315G1&CLASS_ID=34.06&WITH_GENE_MAP=1) |
| SA1898 | sceD | hypothetical protein, 15imilar to SceD precursor | -2.5 | [Pathogenic factors (toxins and colonization factors)](http://www.bio.nite.go.jp/dogan/GeneSearchResult?GENE_LIST_TYPE=1&type=504&GENOME_LIST=n315G1&CLASS_ID=34.06&WITH_GENE_MAP=1) |
| SA0879 | htrA | serine protease HtrA | -2.3 | [Pathogenic factors (toxins and colonization factors)](http://www.bio.nite.go.jp/dogan/GeneSearchResult?GENE_LIST_TYPE=1&type=504&GENOME_LIST=n315G1&CLASS_ID=34.06&WITH_GENE_MAP=1) |
| SA1648 | seo | enterotoxin SeO [Pathogenicity island SaPIn3] | -5.1 | [Pathogenic factors (toxins and colonization factors)](http://www.bio.nite.go.jp/dogan/GeneSearchResult?GENE_LIST_TYPE=1&type=504&GENOME_LIST=n315G1&CLASS_ID=34.06&WITH_GENE_MAP=1) |
| SA1000 |  | hypothetical protein, similar to fibrinogen-binding protein | -4.1 | [Pathogenic factors (toxins and colonization factors)](http://www.bio.nite.go.jp/dogan/GeneSearchResult?GENE_LIST_TYPE=1&type=504&GENOME_LIST=n315G1&CLASS_ID=34.06&WITH_GENE_MAP=1) |
| SA1644 | yent2 | enterotoxin YENT2 [Pathogenicity island SaPIn3] | -3.2 | [Pathogenic factors (toxins and colonization factors)](http://www.bio.nite.go.jp/dogan/GeneSearchResult?GENE_LIST_TYPE=1&type=504&GENOME_LIST=n315G1&CLASS_ID=34.06&WITH_GENE_MAP=1) |
| SA1003 |  | hypothetical protein, similar to fibrinogen-binding protein | -3.9 | [Pathogenic factors (toxins and colonization factors)](http://www.bio.nite.go.jp/dogan/GeneSearchResult?GENE_LIST_TYPE=1&type=504&GENOME_LIST=n315G1&CLASS_ID=34.06&WITH_GENE_MAP=1) |
| SA1751 | truncated(mapW) | truncated map-w protein | -3.9 | [Pathogenic factors (toxins and colonization factors)](http://www.bio.nite.go.jp/dogan/GeneSearchResult?GENE_LIST_TYPE=1&type=504&GENOME_LIST=n315G1&CLASS_ID=34.06&WITH_GENE_MAP=1) |
| SA0309 | geh | glycerol ester hydrolase | -3.6 | [Pathogenic factors (toxins and colonization factors)](http://www.bio.nite.go.jp/dogan/GeneSearchResult?GENE_LIST_TYPE=1&type=504&GENOME_LIST=n315G1&CLASS_ID=34.06&WITH_GENE_MAP=1) |
| SA0901 | sspA | Cysteine protease/V8 protease | -3.8 | [Pathogenic factors (toxins and colonization factors)](http://www.bio.nite.go.jp/dogan/GeneSearchResult?GENE_LIST_TYPE=1&type=504&GENOME_LIST=n315G1&CLASS_ID=34.06&WITH_GENE_MAP=1) |
| SA0620 |  | secretory antigen SsaA homologue | -3.7 | [Pathogenic factors (toxins and colonization factors)](http://www.bio.nite.go.jp/dogan/GeneSearchResult?GENE_LIST_TYPE=1&type=504&GENOME_LIST=n315G1&CLASS_ID=34.06&WITH_GENE_MAP=1) |
| SA2206 | sbi | IgG-binding protein SBI | -5.1 | [Pathogenic factors (toxins and colonization factors)](http://www.bio.nite.go.jp/dogan/GeneSearchResult?GENE_LIST_TYPE=1&type=504&GENOME_LIST=n315G1&CLASS_ID=34.06&WITH_GENE_MAP=1) |
| SA2353 |  | hypothetical protein, similar to secretory antigen precursor SsaA | -6.1 | [Pathogenic factors (toxins and colonization factors)](http://www.bio.nite.go.jp/dogan/GeneSearchResult?GENE_LIST_TYPE=1&type=504&GENOME_LIST=n315G1&CLASS_ID=34.06&WITH_GENE_MAP=1) |
| SA0977 | isdA | cell surface protein | -15.0 | [Pathogenic factors (toxins and colonization factors)](http://www.bio.nite.go.jp/dogan/GeneSearchResult?GENE_LIST_TYPE=1&type=504&GENOME_LIST=n315G1&CLASS_ID=34.06&WITH_GENE_MAP=1) |
| SA2356 | isaA | immunodominant antigen A | -6.2 | [Pathogenic factors (toxins and colonization factors)](http://www.bio.nite.go.jp/dogan/GeneSearchResult?GENE_LIST_TYPE=1&type=504&GENOME_LIST=n315G1&CLASS_ID=34.06&WITH_GENE_MAP=1) |
| SA2093 | ssaA | secretory antigen precursor SsaA homolog | -7.8 | [Pathogenic factors (toxins and colonization factors)](http://www.bio.nite.go.jp/dogan/GeneSearchResult?GENE_LIST_TYPE=1&type=504&GENOME_LIST=n315G1&CLASS_ID=34.06&WITH_GENE_MAP=1) |
| SA0276 |  | conserved hypothetical protein, similar to diarrheal toxin | -7.9 | [Pathogenic factors (toxins and colonization factors)](http://www.bio.nite.go.jp/dogan/GeneSearchResult?GENE_LIST_TYPE=1&type=504&GENOME_LIST=n315G1&CLASS_ID=34.06&WITH_GENE_MAP=1) |
| SA2097 |  | hypothetical protein, similar to secretory antigen precursor SsaA | -9.7 | [Pathogenic factors (toxins and colonization factors)](http://www.bio.nite.go.jp/dogan/GeneSearchResult?GENE_LIST_TYPE=1&type=504&GENOME_LIST=n315G1&CLASS_ID=34.06&WITH_GENE_MAP=1) |
| SA0746 |  | staphylococcal nuclease | -67.0 | [Pathogenic factors (toxins and colonization factors)](http://www.bio.nite.go.jp/dogan/GeneSearchResult?GENE_LIST_TYPE=1&type=504&GENOME_LIST=n315G1&CLASS_ID=34.06&WITH_GENE_MAP=1) |
| SA2207 | hlgA | gamma-hemolysin chain II precursor | 2.5 | [Pathogenic factors (toxins and colonization factors)](http://www.bio.nite.go.jp/dogan/GeneSearchResult?GENE_LIST_TYPE=1&type=504&GENOME_LIST=n315G1&CLASS_ID=34.06&WITH_GENE_MAP=1) |
| SA2006 |  | hypothetical protein, similar to MHC class II analog | 3.5 | [Pathogenic factors (toxins and colonization factors)](http://www.bio.nite.go.jp/dogan/GeneSearchResult?GENE_LIST_TYPE=1&type=504&GENOME_LIST=n315G1&CLASS_ID=34.06&WITH_GENE_MAP=1) |
| SA0742 | clfA | fibrinogen-binding protein A, clumping factor | 5.8 | [Pathogenic factors (toxins and colonization factors)](http://www.bio.nite.go.jp/dogan/GeneSearchResult?GENE_LIST_TYPE=1&type=504&GENOME_LIST=n315G1&CLASS_ID=34.06&WITH_GENE_MAP=1) |
| SA0102 |  | 67 kDa Myosin-crossreactive streptococcal antigen homologue | 4.9 | [Pathogenic factors (toxins and colonization factors)](http://www.bio.nite.go.jp/dogan/GeneSearchResult?GENE_LIST_TYPE=1&type=504&GENOME_LIST=n315G1&CLASS_ID=34.06&WITH_GENE_MAP=1) |
| SAS065 | RNAⅢ | Delta hemolysin | 2.0 | Pathogenic factors(toxins and colonization factors) |
| SA2141 |  | hypothetical protein | -2.2 | No similarity |
| SA0285 |  | hypothetical protein | -2.3 | No similarity |
| SA2091 |  | hypothetical protein | -2.0 | No similarity |
| SA0623 |  | hypothetical protein | -2.4 | No similarity |
| SA0889 |  | hypothetical protein | -2.4 | No similarity |
| SA0535 | vraC | hypothetical protein | -2.4 | No similarity |
| SA0292 |  | hypothetical protein | -2.6 | No similarity |
| SA1514 |  | hypothetical protein | -2.3 | No similarity |
| SA0285 |  | hypothetical protein | -3.1 | No similarity |
| SA2173 |  | hypothetical protein | -2.8 | No similarity |
| SA1665 |  | hypothetical protein | -2.2 | No similarity |
| SA0397 | lpl2 | hypothetical protein [Pathogenicity island SaPIn2] | -2.6 | No similarity |
| SA0396 | lpl1 | hypothetical protein [Pathogenicity island SaPIn2] | -3.4 | No similarity |
| SA1017 |  | hypothetical protein | -3.4 | No similarity |
| SA2274 |  | hypothetical protein | -2.5 | No similarity |
| SA0539 |  | hypothetical protein | -2.6 | No similarity |
| SA1944 |  | hypothetical protein | -2.6 | No similarity |
| SA1620 |  | hypothetical protein | -3.0 | No similarity |
| SA0404 | lpl8 | hypothetical protein [Pathogenicity island SaPIn2] | -3.4 | No similarity |
| SA1056 |  | hypothetical protein | -3.1 | No similarity |
| SA0262 |  | hypothetical protein | -3.7 | No similarity |
| SA0651 |  | hypothetical protein | -3.1 | No similarity |
| SA0364 |  | hypothetical protein | -3.5 | No similarity |
| SA1621 |  | hypothetical protein | -3.2 | No similarity |
| SA0403 | lpl7 | hypothetical protein [Pathogenicity island SaPIn2] | -4.5 | No similarity |
| SA1619 |  | hypothetical protein | -4.1 | No similarity |
| SA0792 |  | hypothetical protein | -5.9 | No similarity |
| SA1726 | scpB | hypothetical protein | -7.1 | No similarity |
| SA0273 |  | hypothetical protein | -7.4 | No similarity |
| SA2126 |  | hypothetical protein | -6.9 | No similarity |
| SA0267 |  | hypothetical protein | -5.8 | No similarity |
| SA0663 |  | hypothetical protein | -8.1 | No similarity |
| SA0268 |  | hypothetical protein | -12.04 | No similarity |
| SA0751 |  | hypothetical protein | 2.2 | No similarity |
| SAS049 |  | hypothetical protein | 2.1 | No similarity |
| SA1567 |  | hypothetical protein | 2.1 | No similarity |
| SA1831 |  | hypothetical protein [Pathogenicity island SaPIn1] | 2.1 | No similarity |
| SA1573 |  | hypothetical protein | 2.0 | No similarity |
| SA2113 |  | hypothetical protein | 2.0 | No similarity |
| SA1822 |  | hypothetical protein [Pathogenicity island SaPIn1] | 2.0 | No similarity |
| SA0931 |  | hypothetical protein | 2.3 | No similarity |
| SA2398 |  | hypothetical protein | 2.5 | No similarity |
| SAS068 |  | hypothetical protein | 3.4 | No similarity |
| SA1821 |  | hypothetical protein [Pathogenicity island SaPIn1] | 2.4 | No similarity |
| SA2292 |  | hypothetical protein | 3.4 | No similarity |
| SAS056 |  | hypothetical protein | 2.7 | No similarity |
| SA2321 |  | hypothetical protein | 2.6 | No similarity |
| SA1567 |  | hypothetical protein | 3.0 | No similarity |
| SA1362 |  | hypothetical protein | 2.7 | No similarity |
| SA2451 |  | hypothetical protein | 2.6 | No similarity |
| SA0955 |  | hypothetical protein | 2.6 | No similarity |
| SA0570 |  | hypothetical protein | 2.6 | No similarity |
| SA1361 |  | hypothetical protein | 3.4 | No similarity |
| SAS056 |  | hypothetical protein | 2.9 | No similarity |
| SA2338 |  | hypothetical protein | 3.3 | No similarity |
| SA0591 |  | hypothetical protein | 2.5 | No similarity |
| SA2321 |  | hypothetical protein | 3.1 | No similarity |
| SA2224 |  | hypothetical protein | 3.1 | No similarity |
| SAS016 |  | hypothetical protein | 3.1 | No similarity |
| SAS037 |  | hypothetical protein | 4.9 | No similarity |
| SA0164 |  | hypothetical protein | 3.1 | No similarity |
| SA1825 |  | hypothetical protein [Pathogenicity island SaPIn1] | 4.3 | No similarity |
| SA0749 |  | hypothetical protein | 3.0 | No similarity |
| SA1824 |  | hypothetical protein [Pathogenicity island SaPIn1] | 3.9 | No similarity |
| SA1823 |  | hypothetical protein | 4.1 | No similarity |
| SA0748 |  | hypothetical protein | 3.6 | No similarity |
| SA2343 |  | hypothetical protein | 10.7 | No similarity |
| SA1476 |  | hypothetical protein | 8.2 | No similarity |
| SA0883 |  | hypothetical protein | 7.8 | No similarity |
| SA1318 | graD | Hypothetical protein | -2.2 | No similarity |
| SA1193 | fmtC/mprF | oxacillin resistance-related FmtC protein | -2.5 | [Miscellaneous](http://www.bio.nite.go.jp/dogan/GeneSearchResult?GENE_LIST_TYPE=1&type=504&GENOME_LIST=n315G1&CLASS_ID=34.07&WITH_GENE_MAP=1) |
| SA0530 |  | hypothetical protein, similar to indigoidine systhesis protein | -3.7 | [Miscellaneous](http://www.bio.nite.go.jp/dogan/GeneSearchResult?GENE_LIST_TYPE=1&type=504&GENOME_LIST=n315G1&CLASS_ID=34.07&WITH_GENE_MAP=1) |
| SA0914 |  | hypothetical protein, similar to chitinase B | 2.3 | [Miscellaneous](http://www.bio.nite.go.jp/dogan/GeneSearchResult?GENE_LIST_TYPE=1&type=504&GENOME_LIST=n315G1&CLASS_ID=34.07&WITH_GENE_MAP=1) |
| SA0231 |  | hypothetical protein, similar to flavohemoprotein | 2.4 | [Miscellaneous](http://www.bio.nite.go.jp/dogan/GeneSearchResult?GENE_LIST_TYPE=1&type=504&GENOME_LIST=n315G1&CLASS_ID=34.07&WITH_GENE_MAP=1) |
| SA0482 |  | hypothetical protein, similar to creatine kinase | 3.7 | [Miscellaneous](http://www.bio.nite.go.jp/dogan/GeneSearchResult?GENE_LIST_TYPE=1&type=504&GENOME_LIST=n315G1&CLASS_ID=34.07&WITH_GENE_MAP=1) |
| SA2413 |  | sulfite reductase (NADPH) (EC 1.8.1.2) flavoprotein | -2.3 | [Metabolism of sulfur](http://www.bio.nite.go.jp/dogan/GeneSearchResult?GENE_LIST_TYPE=1&type=504&GENOME_LIST=n315G1&CLASS_ID=32.07&WITH_GENE_MAP=1) |
| SA0514 |  | hypothetical protein, similar to deoxypurine kinase | -2.2 | [Metabolism of nucleotides and nucleic acids](http://www.bio.nite.go.jp/dogan/GeneSearchResult?GENE_LIST_TYPE=1&type=504&GENOME_LIST=n315G1&CLASS_ID=32.03&WITH_GENE_MAP=1) |
| SA0515 |  | hypothetical protein, similar to deoxypurine kinase | -2.3 | [Metabolism of nucleotides and nucleic acids](http://www.bio.nite.go.jp/dogan/GeneSearchResult?GENE_LIST_TYPE=1&type=504&GENOME_LIST=n315G1&CLASS_ID=32.03&WITH_GENE_MAP=1) |
| SA1013 |  | hypothetical protein, similar to carbamate kinase | -2.3 | [Metabolism of nucleotides and nucleic acids](http://www.bio.nite.go.jp/dogan/GeneSearchResult?GENE_LIST_TYPE=1&type=504&GENOME_LIST=n315G1&CLASS_ID=32.03&WITH_GENE_MAP=1) |
| SA0373 | xprT | xanthine phosphoribosyltransferase | -5.1 | [Metabolism of nucleotides and nucleic acids](http://www.bio.nite.go.jp/dogan/GeneSearchResult?GENE_LIST_TYPE=1&type=504&GENOME_LIST=n315G1&CLASS_ID=32.03&WITH_GENE_MAP=1) |
| SA0022 |  | hypothetical protein, similar to 5’-nucleotidase | -5.8 | [Metabolism of nucleotides and nucleic acids](http://www.bio.nite.go.jp/dogan/GeneSearchResult?GENE_LIST_TYPE=1&type=504&GENOME_LIST=n315G1&CLASS_ID=32.03&WITH_GENE_MAP=1) |
| SA0646 |  | hypothetical protein, similar to deoxyribodipyrimidine photolyase | 2.0 | [Metabolism of nucleotides and nucleic acids](http://www.bio.nite.go.jp/dogan/GeneSearchResult?GENE_LIST_TYPE=1&type=504&GENOME_LIST=n315G1&CLASS_ID=32.03&WITH_GENE_MAP=1) |
| SA0816 |  | hypothetical protein, similar to polyribonucleotide nucleotidyltransferase | 3.2 | [Metabolism of nucleotides and nucleic acids](http://www.bio.nite.go.jp/dogan/GeneSearchResult?GENE_LIST_TYPE=1&type=504&GENOME_LIST=n315G1&CLASS_ID=32.03&WITH_GENE_MAP=1) |
| SA0511 |  | hypothetical protein, similar to UDP-glucose 4-epimerase related protein | -2.2 | [Metabolism of nucleotides and nucleic acids](http://www.bio.nite.go.jp/dogan/GeneSearchResult?GENE_LIST_TYPE=1&type=504&GENOME_LIST=n315G1&CLASS_ID=32.03&WITH_GENE_MAP=1) |
| SA1929 | ctrA | CTP synthase | -2.3 | [Metabolism of nucleotides and nucleic acids](http://www.bio.nite.go.jp/dogan/GeneSearchResult?GENE_LIST_TYPE=1&type=504&GENOME_LIST=n315G1&CLASS_ID=32.03&WITH_GENE_MAP=1) |
| SA0375 | guaB | inositol-monophosphate dehydrogenase | -2.3 | [Metabolism of nucleotides and nucleic acids](http://www.bio.nite.go.jp/dogan/GeneSearchResult?GENE_LIST_TYPE=1&type=504&GENOME_LIST=n315G1&CLASS_ID=32.03&WITH_GENE_MAP=1) |
| SA1160 | nuc | thermonuclease | -2.9 | [Metabolism of nucleotides and nucleic acids](http://www.bio.nite.go.jp/dogan/GeneSearchResult?GENE_LIST_TYPE=1&type=504&GENOME_LIST=n315G1&CLASS_ID=32.03&WITH_GENE_MAP=1) |
| SA1101 | smbA | uridylate kinase | -2.4 | [Metabolism of nucleotides and nucleic acids](http://www.bio.nite.go.jp/dogan/GeneSearchResult?GENE_LIST_TYPE=1&type=504&GENOME_LIST=n315G1&CLASS_ID=32.03&WITH_GENE_MAP=1) |
| SA0376 | guaA | GMP synthase (glutamine-hydrolyzing) | -2.4 | [Metabolism of nucleotides and nucleic acids](http://www.bio.nite.go.jp/dogan/GeneSearchResult?GENE_LIST_TYPE=1&type=504&GENOME_LIST=n315G1&CLASS_ID=32.03&WITH_GENE_MAP=1) |
| SA0022 |  | hypothetical protein, similar to 5’-nucleotidase | -4.1 | [Metabolism of nucleotides and nucleic acids](http://www.bio.nite.go.jp/dogan/GeneSearchResult?GENE_LIST_TYPE=1&type=504&GENOME_LIST=n315G1&CLASS_ID=32.03&WITH_GENE_MAP=1) |
| SA1461 | apt | adenine phosphoribosyl transferase | -2.9 | [Metabolism of nucleotides and nucleic acids](http://www.bio.nite.go.jp/dogan/GeneSearchResult?GENE_LIST_TYPE=1&type=504&GENOME_LIST=n315G1&CLASS_ID=32.03&WITH_GENE_MAP=1) |
| SA1301 | ndk | nucleoside diphosphate kinase | -3.7 | [Metabolism of nucleotides and nucleic acids](http://www.bio.nite.go.jp/dogan/GeneSearchResult?GENE_LIST_TYPE=1&type=504&GENOME_LIST=n315G1&CLASS_ID=32.03&WITH_GENE_MAP=1) |
| SA2078 |  | hypothetical protein, similar to inosine-adenosine-guanosine-nucleoside hydrolase; IAG-nucleoside hydrolase | -3.9 | [Metabolism of nucleotides and nucleic acids](http://www.bio.nite.go.jp/dogan/GeneSearchResult?GENE_LIST_TYPE=1&type=504&GENOME_LIST=n315G1&CLASS_ID=32.03&WITH_GENE_MAP=1) |
| SA0022 |  | hypothetical protein, similar to 5’-nucleotidase | -6.5 | [Metabolism of nucleotides and nucleic acids](http://www.bio.nite.go.jp/dogan/GeneSearchResult?GENE_LIST_TYPE=1&type=504&GENOME_LIST=n315G1&CLASS_ID=32.03&WITH_GENE_MAP=1) |
| SA1172 |  | hypothetical protein, similar to GMP reductase | -12.0 | [Metabolism of nucleotides and nucleic acids](http://www.bio.nite.go.jp/dogan/GeneSearchResult?GENE_LIST_TYPE=1&type=504&GENOME_LIST=n315G1&CLASS_ID=32.03&WITH_GENE_MAP=1) |
| SA0016 | purA | adenylosuccinate synthase | -8.1 | [Metabolism of nucleotides and nucleic acids](http://www.bio.nite.go.jp/dogan/GeneSearchResult?GENE_LIST_TYPE=1&type=504&GENOME_LIST=n315G1&CLASS_ID=32.03&WITH_GENE_MAP=1) |
| SA0131 | pnp | purine nucleoside phosphorylase | 7.9 | [Metabolism of nucleotides and nucleic acids](http://www.bio.nite.go.jp/dogan/GeneSearchResult?GENE_LIST_TYPE=1&type=504&GENOME_LIST=n315G1&CLASS_ID=32.03&WITH_GENE_MAP=1) |
| SA0220 |  | hypothetical protein, similar to glycerophosphodiester phosphodiesterase | -2.2 | [Metabolism of lipids](http://www.bio.nite.go.jp/dogan/GeneSearchResult?GENE_LIST_TYPE=1&type=504&GENOME_LIST=n315G1&CLASS_ID=32.04&WITH_GENE_MAP=1) |
| SA0842 | FabH | 3-oxoacyl-(acyl-carrier protein) synthase homologue | -2.0 | [Metabolism of lipids](http://www.bio.nite.go.jp/dogan/GeneSearchResult?GENE_LIST_TYPE=1&type=504&GENOME_LIST=n315G1&CLASS_ID=32.04&WITH_GENE_MAP=1) |
| SA2333 | mvaA | hydroxymethylglutaryl-CoA reductase | -2.3 | [Metabolism of lipids](http://www.bio.nite.go.jp/dogan/GeneSearchResult?GENE_LIST_TYPE=1&type=504&GENOME_LIST=n315G1&CLASS_ID=32.04&WITH_GENE_MAP=1) |
| SA2080 |  | hypothetical protein, similar to butyryl-CoA dehydrogenase | -2.8 | [Metabolism of lipids](http://www.bio.nite.go.jp/dogan/GeneSearchResult?GENE_LIST_TYPE=1&type=504&GENOME_LIST=n315G1&CLASS_ID=32.04&WITH_GENE_MAP=1) |
| SA1548 |  | hypothetical protein, similar to acylglycerol-3-phosphate O-acyltransfera homolog | -3.0 | [Metabolism of lipids](http://www.bio.nite.go.jp/dogan/GeneSearchResult?GENE_LIST_TYPE=1&type=504&GENOME_LIST=n315G1&CLASS_ID=32.04&WITH_GENE_MAP=1) |
| SA1104 | cdsA | phosphatidate cytidylyltransferase | -3.5 | [Metabolism of lipids](http://www.bio.nite.go.jp/dogan/GeneSearchResult?GENE_LIST_TYPE=1&type=504&GENOME_LIST=n315G1&CLASS_ID=32.04&WITH_GENE_MAP=1) |
| SA0820 | glpQ | glycerophosphoryl diester phosphodiesterase | -7.0 | [Metabolism of lipids](http://www.bio.nite.go.jp/dogan/GeneSearchResult?GENE_LIST_TYPE=1&type=504&GENOME_LIST=n315G1&CLASS_ID=32.04&WITH_GENE_MAP=1) |
| SA2240 |  | hypothetical protein, similar to para-nitrobenzyl esterase chain A | 2.6 | [Metabolism of lipids](http://www.bio.nite.go.jp/dogan/GeneSearchResult?GENE_LIST_TYPE=1&type=504&GENOME_LIST=n315G1&CLASS_ID=32.04&WITH_GENE_MAP=1) |
| SA0473 | folB | 7,8-dihydroneopterin aldolase | -2.7 | [Metabolism of coenzymes and prosthetic groups](http://www.bio.nite.go.jp/dogan/GeneSearchResult?GENE_LIST_TYPE=1&type=504&GENOME_LIST=n315G1&CLASS_ID=32.05&WITH_GENE_MAP=1) |
| SA1586 | ribH | 6,7-dimethyl-8-ribityllumazine synthase | -4.0 | [Metabolism of coenzymes and prosthetic groups](http://www.bio.nite.go.jp/dogan/GeneSearchResult?GENE_LIST_TYPE=1&type=504&GENOME_LIST=n315G1&CLASS_ID=32.05&WITH_GENE_MAP=1) |
| SA1538 |  | hypothetical protein, similar to iron-sulfur cofactor synthesis protein nifZ | -2.1 | [Metabolism of coenzymes and prosthetic groups](http://www.bio.nite.go.jp/dogan/GeneSearchResult?GENE_LIST_TYPE=1&type=504&GENOME_LIST=n315G1&CLASS_ID=32.05&WITH_GENE_MAP=1) |
| SA0894 |  | hypothetical protein, similar to 1,4-dihydroxy-2-naphthodate octaprenyltransferase | -2.3 | [Metabolism of coenzymes and prosthetic groups](http://www.bio.nite.go.jp/dogan/GeneSearchResult?GENE_LIST_TYPE=1&type=504&GENOME_LIST=n315G1&CLASS_ID=32.05&WITH_GENE_MAP=1) |
| SA0472 | folP | dihydropteroate synthase chain A synthetase | -2.3 | [Metabolism of coenzymes and prosthetic groups](http://www.bio.nite.go.jp/dogan/GeneSearchResult?GENE_LIST_TYPE=1&type=504&GENOME_LIST=n315G1&CLASS_ID=32.05&WITH_GENE_MAP=1) |
| SA1494 | hemC | porphobilinogen deaminase | -2.2 | [Metabolism of coenzymes and prosthetic groups](http://www.bio.nite.go.jp/dogan/GeneSearchResult?GENE_LIST_TYPE=1&type=504&GENOME_LIST=n315G1&CLASS_ID=32.05&WITH_GENE_MAP=1) |
| SA1537 |  | hypothetical protein, similar to thiamine biosynthesis protein ThiI | -2.6 | [Metabolism of coenzymes and prosthetic groups](http://www.bio.nite.go.jp/dogan/GeneSearchResult?GENE_LIST_TYPE=1&type=504&GENOME_LIST=n315G1&CLASS_ID=32.05&WITH_GENE_MAP=1) |
| SA1495 | hemX | hemA concentration negative effector hemX | -2.5 | [Metabolism of coenzymes and prosthetic groups](http://www.bio.nite.go.jp/dogan/GeneSearchResult?GENE_LIST_TYPE=1&type=504&GENOME_LIST=n315G1&CLASS_ID=32.05&WITH_GENE_MAP=1) |
| SA0474 | ape | 2-amino-4-hydroxy-6-hydroxymethyldihydropteridine pyrophosphokinase | -2.4 | [Metabolism of coenzymes and prosthetic groups](http://www.bio.nite.go.jp/dogan/GeneSearchResult?GENE_LIST_TYPE=1&type=504&GENOME_LIST=n315G1&CLASS_ID=32.05&WITH_GENE_MAP=1) |
| SA1493 | hemD | uroporphyrinogen III synthase | -2.2 | [Metabolism of coenzymes and prosthetic groups](http://www.bio.nite.go.jp/dogan/GeneSearchResult?GENE_LIST_TYPE=1&type=504&GENOME_LIST=n315G1&CLASS_ID=32.05&WITH_GENE_MAP=1) |
| SA2412 |  | hypothetical protein, similar to uroporphyrin-III C-methyltransferase | -3.0 | [Metabolism of coenzymes and prosthetic groups](http://www.bio.nite.go.jp/dogan/GeneSearchResult?GENE_LIST_TYPE=1&type=504&GENOME_LIST=n315G1&CLASS_ID=32.05&WITH_GENE_MAP=1) |
| SA1919 |  | hypothetical protein, 22ape22cy to protoporphyrinogen oxidase (hemK) | -2.7 | [Metabolism of coenzymes and prosthetic groups](http://www.bio.nite.go.jp/dogan/GeneSearchResult?GENE_LIST_TYPE=1&type=504&GENOME_LIST=n315G1&CLASS_ID=32.05&WITH_GENE_MAP=1) |
| SA1588 | ap | riboflavin synthase alpha chain | -3.6 | [Metabolism of coenzymes and prosthetic groups](http://www.bio.nite.go.jp/dogan/GeneSearchResult?GENE_LIST_TYPE=1&type=504&GENOME_LIST=n315G1&CLASS_ID=32.05&WITH_GENE_MAP=1) |
| SA2077 |  | hypothetical protein, similar to biotin biosynthesis protein | -3.8 | [Metabolism of coenzymes and prosthetic groups](http://www.bio.nite.go.jp/dogan/GeneSearchResult?GENE_LIST_TYPE=1&type=504&GENOME_LIST=n315G1&CLASS_ID=32.05&WITH_GENE_MAP=1) |
| SA1587 | ribA | riboflavin biosynthesis protein | -3.8 | [Metabolism of coenzymes and prosthetic groups](http://www.bio.nite.go.jp/dogan/GeneSearchResult?GENE_LIST_TYPE=1&type=504&GENOME_LIST=n315G1&CLASS_ID=32.05&WITH_GENE_MAP=1) |
| SA1589 | ribD | riboflavin specific deaminase | -2.1 | [Metabolism of coenzymes and prosthetic groups](http://www.bio.nite.go.jp/dogan/GeneSearchResult?GENE_LIST_TYPE=1&type=504&GENOME_LIST=n315G1&CLASS_ID=32.05&WITH_GENE_MAP=1) |
| SA1177 | tkt | transketolase | 2.1 | [Metabolism of carbohydrates and related molecules](http://www.bio.nite.go.jp/dogan/GeneSearchResult?GENE_LIST_TYPE=1&type=504&GENOME_LIST=n315G1&CLASS_ID=32.01&WITH_GENE_MAP=1) |
| SA2327 | cidC | hypothetical protein, similar to pyruvate oxidase | 3.4 | [Metabolism of carbohydrates and related molecules](http://www.bio.nite.go.jp/dogan/GeneSearchResult?GENE_LIST_TYPE=1&type=504&GENOME_LIST=n315G1&CLASS_ID=32.01&WITH_GENE_MAP=1) |
| SA2312 | ddh | D-specific D-2-hydroxyacid dehydrogenase | -2.2 | [Metabolism of carbohydrates and related molecules](http://www.bio.nite.go.jp/dogan/GeneSearchResult?GENE_LIST_TYPE=1&type=504&GENOME_LIST=n315G1&CLASS_ID=32.01&WITH_GENE_MAP=1) |
| SA0791 |  | hypothetical protein, similar to glycerate dehydrogenase | -2.1 | [Metabolism of carbohydrates and related molecules](http://www.bio.nite.go.jp/dogan/GeneSearchResult?GENE_LIST_TYPE=1&type=504&GENOME_LIST=n315G1&CLASS_ID=32.01&WITH_GENE_MAP=1) |
| SA0433 |  | alpha-glucosidase | -2.0 | [Metabolism of carbohydrates and related molecules](http://www.bio.nite.go.jp/dogan/GeneSearchResult?GENE_LIST_TYPE=1&type=504&GENOME_LIST=n315G1&CLASS_ID=32.01&WITH_GENE_MAP=1) |
| SA2007 |  | hypothetical protein, similar to alpha-acetolactate decarboxylase | -2.1 | [Metabolism of carbohydrates and related molecules](http://www.bio.nite.go.jp/dogan/GeneSearchResult?GENE_LIST_TYPE=1&type=504&GENOME_LIST=n315G1&CLASS_ID=32.01&WITH_GENE_MAP=1) |
| SA1724 | purB | adenylosuccinate lyase | -2.3 | [Metabolism of carbohydrates and related molecules](http://www.bio.nite.go.jp/dogan/GeneSearchResult?GENE_LIST_TYPE=1&type=504&GENOME_LIST=n315G1&CLASS_ID=32.01&WITH_GENE_MAP=1) |
| SA0790 |  | hypothetical protein, similar to N-acetyl-glucosamine catabolism homologue | -2.8 | [Metabolism of carbohydrates and related molecules](http://www.bio.nite.go.jp/dogan/GeneSearchResult?GENE_LIST_TYPE=1&type=504&GENOME_LIST=n315G1&CLASS_ID=32.01&WITH_GENE_MAP=1) |
| SA0232 | lctE | L-lactate dehydrogenase | -3.5 | [Metabolism of carbohydrates and related molecules](http://www.bio.nite.go.jp/dogan/GeneSearchResult?GENE_LIST_TYPE=1&type=504&GENOME_LIST=n315G1&CLASS_ID=32.01&WITH_GENE_MAP=1) |
| SA2008 | alsS | alpha-acetolactate synthase | -3.9 | [Metabolism of carbohydrates and related molecules](http://www.bio.nite.go.jp/dogan/GeneSearchResult?GENE_LIST_TYPE=1&type=504&GENOME_LIST=n315G1&CLASS_ID=32.01&WITH_GENE_MAP=1) |
| SA2402 |  | acetate-CoA ligase (EC 6.2.1.1) | 2.1 | [Metabolism of carbohydrates and related molecules](http://www.bio.nite.go.jp/dogan/GeneSearchResult?GENE_LIST_TYPE=1&type=504&GENOME_LIST=n315G1&CLASS_ID=32.01&WITH_GENE_MAP=1) |
| SA1963 | mtlD | mannitol-1-phosphate 5-dehydrogenase | 2.0 | [Metabolism of carbohydrates and related molecules](http://www.bio.nite.go.jp/dogan/GeneSearchResult?GENE_LIST_TYPE=1&type=504&GENOME_LIST=n315G1&CLASS_ID=32.01&WITH_GENE_MAP=1) |
| SA0219 | pflA | formate acetyltransferase activating enzyme | 2.3 | [Metabolism of carbohydrates and related molecules](http://www.bio.nite.go.jp/dogan/GeneSearchResult?GENE_LIST_TYPE=1&type=504&GENOME_LIST=n315G1&CLASS_ID=32.01&WITH_GENE_MAP=1) |
| SA2260 |  | hypothetical protein, similar to glucose 1-dehydrogenase | 2.1 | [Metabolism of carbohydrates and related molecules](http://www.bio.nite.go.jp/dogan/GeneSearchResult?GENE_LIST_TYPE=1&type=504&GENOME_LIST=n315G1&CLASS_ID=32.01&WITH_GENE_MAP=1) |
| SA2327 |  | hypothetical protein, similar to pyruvate oxidase | 2.8 | [Metabolism of carbohydrates and related molecules](http://www.bio.nite.go.jp/dogan/GeneSearchResult?GENE_LIST_TYPE=1&type=504&GENOME_LIST=n315G1&CLASS_ID=32.01&WITH_GENE_MAP=1) |
| SA2119 |  | hypothetical protein, 23ape23cy to dehydrogenase | 2.6 | [Metabolism of carbohydrates and related molecules](http://www.bio.nite.go.jp/dogan/GeneSearchResult?GENE_LIST_TYPE=1&type=504&GENOME_LIST=n315G1&CLASS_ID=32.01&WITH_GENE_MAP=1) |
| SA2346 |  | hypothetical protein, similar to D-specific D-2-hydroxyacid dehydrogenase ddh homolog | 3.2 | [Metabolism of carbohydrates and related molecules](http://www.bio.nite.go.jp/dogan/GeneSearchResult?GENE_LIST_TYPE=1&type=504&GENOME_LIST=n315G1&CLASS_ID=32.01&WITH_GENE_MAP=1) |
| SA2294 | gntK | gluconokinase | 4.8 | [Metabolism of carbohydrates and related molecules](http://www.bio.nite.go.jp/dogan/GeneSearchResult?GENE_LIST_TYPE=1&type=504&GENOME_LIST=n315G1&CLASS_ID=32.01&WITH_GENE_MAP=1) |
| SA0122 | butA | acetoin reductase | 9.5 | [Metabolism of carbohydrates and related molecules](http://www.bio.nite.go.jp/dogan/GeneSearchResult?GENE_LIST_TYPE=1&type=504&GENOME_LIST=n315G1&CLASS_ID=32.01&WITH_GENE_MAP=1) |
| SA1271 |  | threonine deaminase IlvA homolog | -2.7 | [Metabolism of amino acids and related molecules](http://www.bio.nite.go.jp/dogan/GeneSearchResult?GENE_LIST_TYPE=1&type=504&GENOME_LIST=n315G1&CLASS_ID=32.02&WITH_GENE_MAP=1) |
| SA1297 | aroA | 3-phosphoshikimate 1-carboxyvinyltransferase | -2.6 | Metabolism of amino acids and related molecules |
| SA1012 | argF | ornithine carbamoyltransferase | -3.6 | Metabolism of amino acids and related molecules |
| SA2121 | hutI | imidazolonepropionase | -3.2 | Metabolism of amino acids and related molecules |
| SA2122 | hutU | urocanate hydratase | -4.1 | Metabolism of amino acids and related molecules |
| SA0008 | hutH | histidine ammonia-lyase | -11.7 | Metabolism of amino acids and related molecules |
| SA2088 | ureD | urease accessory protein UreD | 2.0 | [Metabolism of amino acids and related molecules](http://www.bio.nite.go.jp/dogan/GeneSearchResult?GENE_LIST_TYPE=1&type=504&GENOME_LIST=n315G1&CLASS_ID=32.02&WITH_GENE_MAP=1) |
| SA0011 |  | hypothetical protein, similar to homoserine-o-acetyltransferase | 2.0 | [Metabolism of amino acids and related molecules](http://www.bio.nite.go.jp/dogan/GeneSearchResult?GENE_LIST_TYPE=1&type=504&GENOME_LIST=n315G1&CLASS_ID=32.02&WITH_GENE_MAP=1) |
| SA1959 | glmS | glucosamine-fructose-6-phosphate aminotransferase | 2.1 | [Metabolism of amino acids and related molecules](http://www.bio.nite.go.jp/dogan/GeneSearchResult?GENE_LIST_TYPE=1&type=504&GENOME_LIST=n315G1&CLASS_ID=32.02&WITH_GENE_MAP=1) |
| SA2084 | ureC | urease alpha subunit | 2.1 | [Metabolism of amino acids and related molecules](http://www.bio.nite.go.jp/dogan/GeneSearchResult?GENE_LIST_TYPE=1&type=504&GENOME_LIST=n315G1&CLASS_ID=32.02&WITH_GENE_MAP=1) |
| SA2465 | hisF | cyclase-like protein hisF | 2.1 | [Metabolism of amino acids and related molecules](http://www.bio.nite.go.jp/dogan/GeneSearchResult?GENE_LIST_TYPE=1&type=504&GENOME_LIST=n315G1&CLASS_ID=32.02&WITH_GENE_MAP=1) |
| SA1227 | dapA | dihydrodipicolinate synthase | 2.2 | [Metabolism of amino acids and related molecules](http://www.bio.nite.go.jp/dogan/GeneSearchResult?GENE_LIST_TYPE=1&type=504&GENOME_LIST=n315G1&CLASS_ID=32.02&WITH_GENE_MAP=1) |
| SA1225 | lysC | aspartokinase II | 2.5 | [Metabolism of amino acids and related molecules](http://www.bio.nite.go.jp/dogan/GeneSearchResult?GENE_LIST_TYPE=1&type=504&GENOME_LIST=n315G1&CLASS_ID=32.02&WITH_GENE_MAP=1) |
| SA1861 | ilvC | alpha-keto-beta-hydroxylacil reductoisomerase | 3.9 | [Metabolism of amino acids and related molecules](http://www.bio.nite.go.jp/dogan/GeneSearchResult?GENE_LIST_TYPE=1&type=504&GENOME_LIST=n315G1&CLASS_ID=32.02&WITH_GENE_MAP=1) |
| SA2347 |  | hypothetical protein, similar to aspartate aminotransferase | 3.4 | [Metabolism of amino acids and related molecules](http://www.bio.nite.go.jp/dogan/GeneSearchResult?GENE_LIST_TYPE=1&type=504&GENOME_LIST=n315G1&CLASS_ID=32.02&WITH_GENE_MAP=1) |
| SA0180 |  | hypothetical protein, 24ape24cy to branched-chain amino acid transport system carrier protein | -2.1 | Metabolism of amino acids and related molecules |
| SA1298 | aroB | 3-dehydroquinate synthase | -2.5 | Metabolism of amino acids and related molecules |
| SA2319 |  | hypothetical protein, similar to beta-subunit of L-serine dehydratas | -2.9 | Metabolism of amino acids and related molecules |
| SA2318 |  | hypothetical protein, similar to L-serine dehydratase | -2.0 | Metabolism of amino acids and related molecules |
| SA1608 | metK | S-adenosylmethionine synthetase | -2.4 | Metabolism of amino acids and related molecules |
| SA0822 | argG | argininosuccinate synthase | -2.4 | Metabolism of amino acids and related molecules |
| SA0859 |  | thimet oligopeptidase homologue | -2.4 | Metabolism of amino acids and related molecules |
| SA2099 |  | hypothetical protein, similar to monooxygenase | -3.2 | Metabolism of amino acids and related molecules |
| SA1310 | ansA | probable L-asparaginase | -3.9 | Metabolism of amino acids and related molecules |
| SA1272 |  | alanine dehydrogenase | -4.9 | Metabolism of amino acids and related molecules |
| SA2125 |  | hypothetical protein, similar to formiminoglutamase | 2.1 | Metabolism of amino acids and related molecules |
| SA1858 | ilvD | dihydroxy-acid dehydratase | 2.2 | [Metabolism of amino acids and related molecules](http://www.bio.nite.go.jp/dogan/GeneSearchResult?GENE_LIST_TYPE=1&type=504&GENOME_LIST=n315G1&CLASS_ID=32.02&WITH_GENE_MAP=1) |
| SA1204 | trpB | tryptophan synthase beta chain | 2.2 | [Metabolism of amino acids and related molecules](http://www.bio.nite.go.jp/dogan/GeneSearchResult?GENE_LIST_TYPE=1&type=504&GENOME_LIST=n315G1&CLASS_ID=32.02&WITH_GENE_MAP=1) |
| SA1226 | asd | aspartate semialdehyde dehydrogenase | 2.2 | [Metabolism of amino acids and related molecules](http://www.bio.nite.go.jp/dogan/GeneSearchResult?GENE_LIST_TYPE=1&type=504&GENOME_LIST=n315G1&CLASS_ID=32.02&WITH_GENE_MAP=1) |
| SA1864 | leuC | 3-isopropylmalate dehydratase large subunit | 2.1 | [Metabolism of amino acids and related molecules](http://www.bio.nite.go.jp/dogan/GeneSearchResult?GENE_LIST_TYPE=1&type=504&GENOME_LIST=n315G1&CLASS_ID=32.02&WITH_GENE_MAP=1) |
| SA1228 | dapB | dihydrodipicolinate reductase | 2.3 | [Metabolism of amino acids and related molecules](http://www.bio.nite.go.jp/dogan/GeneSearchResult?GENE_LIST_TYPE=1&type=504&GENOME_LIST=n315G1&CLASS_ID=32.02&WITH_GENE_MAP=1) |
| SA2397 |  | hypothetical protein, similar to pyridoxal-phosphate dependent aminotransferase | 3.3 | [Metabolism of amino acids and related molecules](http://www.bio.nite.go.jp/dogan/GeneSearchResult?GENE_LIST_TYPE=1&type=504&GENOME_LIST=n315G1&CLASS_ID=32.02&WITH_GENE_MAP=1) |
| SA2464 | hisI | histidine biosynthesis bifunctional protein HisIE | 2.1 | [Metabolism of amino acids and related molecules](http://www.bio.nite.go.jp/dogan/GeneSearchResult?GENE_LIST_TYPE=1&type=504&GENOME_LIST=n315G1&CLASS_ID=32.02&WITH_GENE_MAP=1) |
| SA1166 | thrB | homoserine kinase homolog | 4.5 | [Metabolism of amino acids and related molecules](http://www.bio.nite.go.jp/dogan/GeneSearchResult?GENE_LIST_TYPE=1&type=504&GENOME_LIST=n315G1&CLASS_ID=32.02&WITH_GENE_MAP=1) |
| SA1863 | leuB | 3-isopropylmalate dehydrogenase | 2.8 | [Metabolism of amino acids and related molecules](http://www.bio.nite.go.jp/dogan/GeneSearchResult?GENE_LIST_TYPE=1&type=504&GENOME_LIST=n315G1&CLASS_ID=32.02&WITH_GENE_MAP=1) |
| SA1860 |  | hypothetical protein, similar to acetolactate synthase small subunit | 4.6 | [Metabolism of amino acids and related molecules](http://www.bio.nite.go.jp/dogan/GeneSearchResult?GENE_LIST_TYPE=1&type=504&GENOME_LIST=n315G1&CLASS_ID=32.02&WITH_GENE_MAP=1) |
| SA1859 | ilvB | acetolactate synthase large subunit | 4.0 | [Metabolism of amino acids and related molecules](http://www.bio.nite.go.jp/dogan/GeneSearchResult?GENE_LIST_TYPE=1&type=504&GENOME_LIST=n315G1&CLASS_ID=32.02&WITH_GENE_MAP=1) |
| SA1163 |  | aspartate kinase homolog | 4.4 | [Metabolism of amino acids and related molecules](http://www.bio.nite.go.jp/dogan/GeneSearchResult?GENE_LIST_TYPE=1&type=504&GENOME_LIST=n315G1&CLASS_ID=32.02&WITH_GENE_MAP=1) |
| SA1164 | dhoM | homoserine dehydrogenase | 4.1 | [Metabolism of amino acids and related molecules](http://www.bio.nite.go.jp/dogan/GeneSearchResult?GENE_LIST_TYPE=1&type=504&GENOME_LIST=n315G1&CLASS_ID=32.02&WITH_GENE_MAP=1) |
| SA2427 | arcB | ornithine carbamoyltransferase | 3.8 | [Metabolism of amino acids and related molecules](http://www.bio.nite.go.jp/dogan/GeneSearchResult?GENE_LIST_TYPE=1&type=504&GENOME_LIST=n315G1&CLASS_ID=32.02&WITH_GENE_MAP=1) |
| SA1862 | leuA | 2-isopropylmalate synthase | 3.6 | [Metabolism of amino acids and related molecules](http://www.bio.nite.go.jp/dogan/GeneSearchResult?GENE_LIST_TYPE=1&type=504&GENOME_LIST=n315G1&CLASS_ID=32.02&WITH_GENE_MAP=1) |
| SA2428 | arcA | arginine deiminase | 4.0 | [Metabolism of amino acids and related molecules](http://www.bio.nite.go.jp/dogan/GeneSearchResult?GENE_LIST_TYPE=1&type=504&GENOME_LIST=n315G1&CLASS_ID=32.02&WITH_GENE_MAP=1) |
| SA1165 | thrC | threonine synthase | 4.7 | [Metabolism of amino acids and related molecules](http://www.bio.nite.go.jp/dogan/GeneSearchResult?GENE_LIST_TYPE=1&type=504&GENOME_LIST=n315G1&CLASS_ID=32.02&WITH_GENE_MAP=1) |
| SA0817 |  | hypothetical protein, similar to NADH-dependent flavin oxidoreductase | 2.1 | [Membrane bioenergetics (electron transport chain and ATP synthase)](http://www.bio.nite.go.jp/dogan/GeneSearchResult?GENE_LIST_TYPE=1&type=504&GENOME_LIST=n315G1&CLASS_ID=31.04&WITH_GENE_MAP=1) |
| SA1241 |  | hypothetical protein, similar to nitric-oxide reductase | -2.4 | [Membrane bioenergetics (electron transport chain and ATP synthase)](http://www.bio.nite.go.jp/dogan/GeneSearchResult?GENE_LIST_TYPE=1&type=504&GENOME_LIST=n315G1&CLASS_ID=31.04&WITH_GENE_MAP=1) |
| SA1132 |  | hypothetical protein, similar to 2-oxoacid ferredoxin oxidoreductase, beta subunit | -2.2 | [Membrane bioenergetics (electron transport chain and ATP synthase)](http://www.bio.nite.go.jp/dogan/GeneSearchResult?GENE_LIST_TYPE=1&type=504&GENOME_LIST=n315G1&CLASS_ID=31.04&WITH_GENE_MAP=1) |
| SA1315 | fer | ferredoxin | -2.1 | [Membrane bioenergetics (electron transport chain and ATP synthase)](http://www.bio.nite.go.jp/dogan/GeneSearchResult?GENE_LIST_TYPE=1&type=504&GENOME_LIST=n315G1&CLASS_ID=31.04&WITH_GENE_MAP=1) |
| SA1131 |  | hypothetical protein, similar to 2-oxoacid ferredoxin oxidoreductase, alpha subunit | -2.1 | [Membrane bioenergetics (electron transport chain and ATP synthase)](http://www.bio.nite.go.jp/dogan/GeneSearchResult?GENE_LIST_TYPE=1&type=504&GENOME_LIST=n315G1&CLASS_ID=31.04&WITH_GENE_MAP=1) |
| SA0411 | ndhF | NADH dehydrogenase subunit 5 | 2.0 | [Membrane bioenergetics (electron transport chain and ATP synthase)](http://www.bio.nite.go.jp/dogan/GeneSearchResult?GENE_LIST_TYPE=1&type=504&GENOME_LIST=n315G1&CLASS_ID=31.04&WITH_GENE_MAP=1) |
| SA0367 |  | hypothetical protein, similar to nitro/flavin reductase | 2.1 | [Membrane bioenergetics (electron transport chain and ATP synthase)](http://www.bio.nite.go.jp/dogan/GeneSearchResult?GENE_LIST_TYPE=1&type=504&GENOME_LIST=n315G1&CLASS_ID=31.04&WITH_GENE_MAP=1) |
| SA2324 |  | hypothetical protein, similar to thioredoxin | 2.3 | [Membrane bioenergetics (electron transport chain and ATP synthase)](http://www.bio.nite.go.jp/dogan/GeneSearchResult?GENE_LIST_TYPE=1&type=504&GENOME_LIST=n315G1&CLASS_ID=31.04&WITH_GENE_MAP=1) |
| SA2162 |  |  | 2.7 | [Membrane bioenergetics (electron transport chain and ATP synthase)](http://www.bio.nite.go.jp/dogan/GeneSearchResult?GENE_LIST_TYPE=1&type=504&GENOME_LIST=n315G1&CLASS_ID=31.04&WITH_GENE_MAP=1) |
| SA1989 |  | hypothetical protein, similar to 27ape27cy oxidoreductase | 3.6 | [Membrane bioenergetics (electron transport chain and ATP synthase)](http://www.bio.nite.go.jp/dogan/GeneSearchResult?GENE_LIST_TYPE=1&type=504&GENOME_LIST=n315G1&CLASS_ID=31.04&WITH_GENE_MAP=1) |
| SA1087 | rnhB | Rnase HII | -2.2 | DNA replication |
| SA1720 | lig | DNA ligase (polydeoxyribonucleotide syntase [NAD+]) | -2.1 | DNA replication |
| SA1181 |  | hypothetical protein, similar to exonuclease SbcC | -2.1 | [DNA recombination](http://www.bio.nite.go.jp/dogan/GeneSearchResult?GENE_LIST_TYPE=1&type=504&GENOME_LIST=n315G1&CLASS_ID=33.03&WITH_GENE_MAP=1) |
| SA1462 |  | hypothetical protein, similar to single-strand DNA-specific exonuclease | -3.2 | [DNA recombination](http://www.bio.nite.go.jp/dogan/GeneSearchResult?GENE_LIST_TYPE=1&type=504&GENOME_LIST=n315G1&CLASS_ID=33.03&WITH_GENE_MAP=1) |
| SA1070 | recG | ATP-dependent DNA helicase | 2.1 | [DNA recombination](http://www.bio.nite.go.jp/dogan/GeneSearchResult?GENE_LIST_TYPE=1&type=504&GENOME_LIST=n315G1&CLASS_ID=33.03&WITH_GENE_MAP=1) |
| SA1092 |  | hypothetical protein, similar to DNA processing Smf protein | -3.0 | [DNA packaging and segregation](http://www.bio.nite.go.jp/dogan/GeneSearchResult?GENE_LIST_TYPE=1&type=504&GENOME_LIST=n315G1&CLASS_ID=33.04&WITH_GENE_MAP=1) |
| SA0392 | hsdS | probable restriction modification system specificity subunit [Pathogenicity island SaPIn2] | -2.0 | [DNA modification and repair](http://www.bio.nite.go.jp/dogan/GeneSearchResult?GENE_LIST_TYPE=1&type=504&GENOME_LIST=n315G1&CLASS_ID=33.02&WITH_GENE_MAP=1) |
| SA2278 |  | hypothetical protein, 28ape28cy to mutator protein 28ape | -2.2 | [DNA modification and repair](http://www.bio.nite.go.jp/dogan/GeneSearchResult?GENE_LIST_TYPE=1&type=504&GENOME_LIST=n315G1&CLASS_ID=33.02&WITH_GENE_MAP=1) |
| SA1386 |  | hypothetical protein, similar to endonuclease IV | -2.2 | [DNA modification and repair](http://www.bio.nite.go.jp/dogan/GeneSearchResult?GENE_LIST_TYPE=1&type=504&GENOME_LIST=n315G1&CLASS_ID=33.02&WITH_GENE_MAP=1) |
| SA0391 | hsdM | probable type I site-specific deoxyribonuclease (EC 3.1.21.3) LldI chain hsdM [Pathogenicity island SaPIn2] | -2.9 | [DNA modification and repair](http://www.bio.nite.go.jp/dogan/GeneSearchResult?GENE_LIST_TYPE=1&type=504&GENOME_LIST=n315G1&CLASS_ID=33.02&WITH_GENE_MAP=1) |
| SA0538 | ung | uracil-DNA glycosylase | -3.3 | [DNA modification and repair](http://www.bio.nite.go.jp/dogan/GeneSearchResult?GENE_LIST_TYPE=1&type=504&GENOME_LIST=n315G1&CLASS_ID=33.02&WITH_GENE_MAP=1) |
| SA0189 | hsdR | probable type I restriction enzyme restriction chain | -3.7 | [DNA modification and repair](http://www.bio.nite.go.jp/dogan/GeneSearchResult?GENE_LIST_TYPE=1&type=504&GENOME_LIST=n315G1&CLASS_ID=33.02&WITH_GENE_MAP=1) |
| SA1853 |  | hypothetical protein, ape cy to DNA mismatch repair protein MutS | -6.3 | [DNA modification and repair](http://www.bio.nite.go.jp/dogan/GeneSearchResult?GENE_LIST_TYPE=1&type=504&GENOME_LIST=n315G1&CLASS_ID=33.02&WITH_GENE_MAP=1) |
| SA1711 |  | hypothetical protein, similar to DNA-damage inducible protein P | 4.1 | [DNA modification and repair](http://www.bio.nite.go.jp/dogan/GeneSearchResult?GENE_LIST_TYPE=1&type=504&GENOME_LIST=n315G1&CLASS_ID=33.02&WITH_GENE_MAP=1) |
| SA2317 |  | hypothetical protein, similar to N-acetyltransferase | -2.7 | [Detoxification](http://www.bio.nite.go.jp/dogan/GeneSearchResult?GENE_LIST_TYPE=1&type=504&GENOME_LIST=n315G1&CLASS_ID=34.02&WITH_GENE_MAP=1) |
| SA0128 | sodM | superoxide dismutase | -4.6 | [Detoxification](http://www.bio.nite.go.jp/dogan/GeneSearchResult?GENE_LIST_TYPE=1&type=504&GENOME_LIST=n315G1&CLASS_ID=34.02&WITH_GENE_MAP=1) |
| SA0312 |  | hypothetical protein, similar to alkanal monooxygenase alpha chain | 2.4 | [Detoxification](http://www.bio.nite.go.jp/dogan/GeneSearchResult?GENE_LIST_TYPE=1&type=504&GENOME_LIST=n315G1&CLASS_ID=34.02&WITH_GENE_MAP=1) |
| SA0681 |  | hypothetical protein, similar to multidrug resistance protein | 2.9 | [Detoxification](http://www.bio.nite.go.jp/dogan/GeneSearchResult?GENE_LIST_TYPE=1&type=504&GENOME_LIST=n315G1&CLASS_ID=34.02&WITH_GENE_MAP=1) |
| SA0132 |  | hypothetical protein, similar to 28ape28cycline resistance protein | 3.9 | Detoxification |
| SA1458 | lytH | N-acetylmuramoyl-L-alanine amidase | -3.0 | [Cell wall](http://www.bio.nite.go.jp/dogan/GeneSearchResult?GENE_LIST_TYPE=1&type=504&GENOME_LIST=n315G1&CLASS_ID=31.01&WITH_GENE_MAP=1) |
| SA0795 | dltC | D-Alanine-poly(phosphoribitol) ligase subunit 2 | -4.1 | [Cell wall](http://www.bio.nite.go.jp/dogan/GeneSearchResult?GENE_LIST_TYPE=1&type=504&GENOME_LIST=n315G1&CLASS_ID=31.01&WITH_GENE_MAP=1) |
| SA0875 |  | hypothetical protein, similar to cell wall synthesis protein | 2.2 | [Cell wall](http://www.bio.nite.go.jp/dogan/GeneSearchResult?GENE_LIST_TYPE=1&type=504&GENOME_LIST=n315G1&CLASS_ID=31.01&WITH_GENE_MAP=1) |
| SA2441 |  | Glycosyl transferase, group1 family protein | 2.8 | [Cell wall](http://www.bio.nite.go.jp/dogan/GeneSearchResult?GENE_LIST_TYPE=1&type=504&GENOME_LIST=n315G1&CLASS_ID=31.01&WITH_GENE_MAP=1) |
| SA2288 | gtaB | UTP-glucose-1-phosphate uridyltransferase | -2.2 | [Cell wall](http://www.bio.nite.go.jp/dogan/GeneSearchResult?GENE_LIST_TYPE=1&type=504&GENOME_LIST=n315G1&CLASS_ID=31.01&WITH_GENE_MAP=1) |
| SA1103 | uppS | undecaprenyl diphosphate synthase | -2.8 | [Cell wall](http://www.bio.nite.go.jp/dogan/GeneSearchResult?GENE_LIST_TYPE=1&type=504&GENOME_LIST=n315G1&CLASS_ID=31.01&WITH_GENE_MAP=1) |
| SA2354 |  | hypothetical protein, similar to acyltransferase | -4.2 | [Cell wall](http://www.bio.nite.go.jp/dogan/GeneSearchResult?GENE_LIST_TYPE=1&type=504&GENOME_LIST=n315G1&CLASS_ID=31.01&WITH_GENE_MAP=1) |
| SA0793 | dltA | D-alanine-D-alanyl carrier protein ligase | -4.9 | [Cell wall](http://www.bio.nite.go.jp/dogan/GeneSearchResult?GENE_LIST_TYPE=1&type=504&GENOME_LIST=n315G1&CLASS_ID=31.01&WITH_GENE_MAP=1) |
| SA0423 |  | hypothetical protein, similar to autolysin (N-acetylmuramoyl-L-alanine amidase) | -10.9 | [Cell wall](http://www.bio.nite.go.jp/dogan/GeneSearchResult?GENE_LIST_TYPE=1&type=504&GENOME_LIST=n315G1&CLASS_ID=31.01&WITH_GENE_MAP=1) |
| SA0205 |  | hypothetical protein, similar to lysostaphin precursor | 3.0 | [Cell wall](http://www.bio.nite.go.jp/dogan/GeneSearchResult?GENE_LIST_TYPE=1&type=504&GENOME_LIST=n315G1&CLASS_ID=31.01&WITH_GENE_MAP=1) |
| SA1091 | eprH | Endopeptidase resistance gene | -2.2 | Cell wall |
| SAV1017 ^b^ | ypfP | Hypothetical protein,similar to cell wall synthesis protein | 2.2 | Cell wall |
| SA0456 | spoVG | stage V sporulation protein G homologue | 2.5 | [Cell division](http://www.bio.nite.go.jp/dogan/GeneSearchResult?GENE_LIST_TYPE=1&type=504&GENOME_LIST=n315G1&CLASS_ID=31.07&WITH_GENE_MAP=1) |
| SA0724 |  | hypothetical protein, similar to cell-division inhibitor | 2.9 | [Cell division](http://www.bio.nite.go.jp/dogan/GeneSearchResult?GENE_LIST_TYPE=1&type=504&GENOME_LIST=n315G1&CLASS_ID=31.07&WITH_GENE_MAP=1) |
| SA0905 | atl | Bifunctional precursor autolysin (Atl) | -3.3 | cell division |
| SA2406 | gbsA | glycine betaine aldehyde dehydrogenase gbsA | -13.0 | [Adaption to atypical conditions](http://www.bio.nite.go.jp/dogan/GeneSearchResult?GENE_LIST_TYPE=1&type=504&GENOME_LIST=n315G1&CLASS_ID=34.01&WITH_GENE_MAP=1) |
| SA2405 | ape | choline dehydrogenase | -16.6 | [Adaption to atypical conditions](http://www.bio.nite.go.jp/dogan/GeneSearchResult?GENE_LIST_TYPE=1&type=504&GENOME_LIST=n315G1&CLASS_ID=34.01&WITH_GENE_MAP=1) |
| SA0146 | capC | capsular polysaccharide synthesis enzyme Cap8C | 2.1 | [Adaption to atypical conditions](http://www.bio.nite.go.jp/dogan/GeneSearchResult?GENE_LIST_TYPE=1&type=504&GENOME_LIST=n315G1&CLASS_ID=34.01&WITH_GENE_MAP=1) |
| SA0148 | ape | capsular polysaccharide synthesis enzyme Cap8E | 2.1 | [Adaption to atypical conditions](http://www.bio.nite.go.jp/dogan/GeneSearchResult?GENE_LIST_TYPE=1&type=504&GENOME_LIST=n315G1&CLASS_ID=34.01&WITH_GENE_MAP=1) |
| SA0659 |  | hypothetical protein, similar to CsbB stress response protein | 2.2 | [Adaption to atypical conditions](http://www.bio.nite.go.jp/dogan/GeneSearchResult?GENE_LIST_TYPE=1&type=504&GENOME_LIST=n315G1&CLASS_ID=34.01&WITH_GENE_MAP=1) |
| SA1941 | dps | general stress protein 20U | 2.4 | [Adaption to atypical conditions](http://www.bio.nite.go.jp/dogan/GeneSearchResult?GENE_LIST_TYPE=1&type=504&GENOME_LIST=n315G1&CLASS_ID=34.01&WITH_GENE_MAP=1) |
| SA1410 | grpE | GrpE protein (HSP-70 Cofactor HSP20) | 2.7 | [Adaption to atypical conditions](http://www.bio.nite.go.jp/dogan/GeneSearchResult?GENE_LIST_TYPE=1&type=504&GENOME_LIST=n315G1&CLASS_ID=34.01&WITH_GENE_MAP=1) |
| SA0483 | clpC | endopeptidase | 3.4 | [Adaption to atypical conditions](http://www.bio.nite.go.jp/dogan/GeneSearchResult?GENE_LIST_TYPE=1&type=504&GENOME_LIST=n315G1&CLASS_ID=34.01&WITH_GENE_MAP=1) |
| SA0835 | clpB | ClpB chaperone homologue | 7.8 | [Adaption to atypical conditions](http://www.bio.nite.go.jp/dogan/GeneSearchResult?GENE_LIST_TYPE=1&type=504&GENOME_LIST=n315G1&CLASS_ID=34.01&WITH_GENE_MAP=1) |
