# Supplementary material for: Transcriptional and Functional Analysis of the Effects of Magnolol: Inhibition of Autolysis and Biofilms in Staphylococcus aureus
Source: PLoS One. 2011 Oct 28;6(10):e26833. doi: 10.1371/journal.pone.0026833 (PMC3203910; doi:10.1371/journal.pone.0026833)
Supplement: Table S2 — Genes involved in autolysis or known as related regulators affected by MOL. a − indicates reduction and + indicates increase; NS, not significant. (DOC) [file pone.0026833.s002.doc]

| **N315 SA no.** | **Gene** | **Product or putative function** | **Fold change***^a^* |
| --- | --- | --- | --- |
| **Autolysis genes** |  |  |  |
| SA0905 | *atl* | Bifunctional precursor autolysin (Atl) | -3.3 |
| SA0423 | *sle1* | N-Acetylmuramyl-L-alanine amidase | -10.9 |
| SA0265 | *lytM* | Peptidoglycan hydrolase | NS |
| SA1090 | *lytN* | LytN protein | -1.5 |
| SA2329 | *cidA* | Hypothetical protein, similar to transcription regulator | -3.3 |
| SA2328 | *cidB* | Conserved hypothetical protein | 2.3 |
| SA2327 | *cidC* | Pyruvate oxidase | 3.4 |
| SA0251 | *lytR* | Two-component response regulator | -2.7 |
| SA0250 | *lytS* | Two-component sensor histidine kinase | -1.6 |
| SA0252 | *lrgA* | Murein hydrolase regulator LrgA | 35.0 |
| SA0253 | *lrgB* | Antiholin-like protein LrgB | 30.9 |
| SA0641 | *mgrA* | Transcriptional regulator MgrA | NS |
| SA0650 | *norA* | Quinolone resistance protein | NS |
| SA1248 | *arlR* | Truncated (putative response regulator ArlR | 2.0 |
| SA1246 | *arlS* | Sensor histidine kinase ArlS | NS |
| SA0573 | *sarA* | Staphylococcal accessory regulator A | 3.1 |
| SAS065 | *RNAIII* | Delta hemolysin | 2.,0 |
| SA1844 | *agrA* | Accessory gene regulator A | 2.3 |
| SA0904 |  | Hypothetical protein, probable ATL autolysin transcription regulator | -1.6 |
| SA1091 | *eprH* | Endopeptidase resistance gene | -2.2 |
| SA0702 | *llm/tagO* | Lipophilic protein affecting bacterial lysis rate and methicillin resistance level | NS |
| SA0909 | *fmtA* | Autolysis and methicillin resistant-related protein | Absent |
| SA2062 | *sarV* | Staphylococcal accessory regulator A homolog | NS |
| SA1964 | *fmtB* | FmtB protein | NS |
| SA1028 | *ftsA* | Cell division protein | 1.5 |
| SA1029 | *ftsZ* | Cell division protein | 1.5 |
| SA0249 | *scdA* | Cell division protein and morphogenesis-related protein | NS |
| SA1193 | *fmtC/mprF* | Oxacillin resistance-related FmtC protein | -2.5 |
| SA0793 | *dltA* | D-Alanine-D-alanyl carrier protein ligase | -4.9 |
| SA0794 | *dltB* | DltB membrane protein | -2.6 |
| SA0795 | *dltC* | D-Alanine-poly(phosphoribitol) ligase subunit 2 | -4.1 |
| SA0796 | *dltD* | Poly D-alanine transfer protein | -4.9 |
| SA1898 | *sceD* | Hypothetical protein, simialr to SceD precursor | -2.5 |
| SA2356 | *isaA* | Immunodominant antigen A | -6.2 |
| SA2093 | *ssaA* | Secretory antigen precursor SsaA homolog | -7.8 |
| SA0620 |  | Secretory antigen SsaA homologue | -3.7 |
| SA0710 |  | Hypothetical protein | NS |
| SA2097 |  | Hypothetical protein, similar to secretory antigen precursor SsaA | -9.7 |
| SA2353 |  | Hypothetical protein, similar to secretory antigen precursor SsaA | -6.1 |
| **Global or antibiotic cell wall regulators** |  |  |  |
| SA2145 | *tcaB* | TcaB protein | -3.4 |
| SA2480 | *drp35* | Drp35 | -1.8 |
| SA1194 | *msrA2* | Methionine sulfoxide reductase A | 1.9 |
| SA1195 | *msrR* | Peptide methionine sulfoxide reductase regulator MsrR | NS |
| SA1257 | *msrA1* | Peptide methionine sulfoxide reductase A | NS |
| SA1256 | *msrB* | Methionine sulfoxide reductase B | NS |
| SA2146 | *tcaA* | TcaA protein | -1.7 |
| SA2147 | *tcaR* | TcaR transcription regulator | -1.2 |
| SA1700 | *vraR* | Two-component response regulator | 2.0 |
| SA1701 | *vraS* | Two-component sensor histidine kinase | 1.9 |
| SA1691 | *sgtB* | Monofunctional glycosyltransferase | NS |
| SA1659 | *prsA* | Peptidyl-prolyl cis/trans isomerase homolog | NS |
| SA1551 | *sgtA* | Monofunctional glycosyltransferase | NS |
| SA0599 | *abcA* | ATP-binding cassette transporter A | 1.7 |
| SA1869 | *sigB* | Sigma factor B | NS |
| SA1872 | *rsbU* | Sigma B regulation protein RsbU | -2.3 |
| SA1871 | *rsbV* | Anti-sigma B factor antagonist | NS |
| SA1870 | *rsbW* | Serine-protein kinase RsbW | NS |
| SA1984 | *asp23* | Alkaline shock protein 23, Asp23 | 1.9 |
| SA0108 | *sarS* | Staphylococcal accessory regulator A homologue | -1.8 |
| SA0018 | *yycG* | Two-component sensor histidine kinase | NS |
| SA0017 | *yycF* | Response regulator | NS |
| SA1323 | *srrA* | Staphylococcal respiratory response protein SrrA | NS |
| SA1322 | *srrB* | Staphylococcal respiratory response protein SrrB | NS |
| SA0661 | *saeR* | Response regulator | -1.5 |
| SA0660 | *saeS* | Histidine protein kinase | -1.5 |
| SA1583 | *rot* | Repressor of toxins Rot | NS |
| SA2287 | *sarU* | Staphylococcal accessory regulator A homolog | Absent |
| SA2286 | *sarT* | Staphylococcal accessory regulator A homolog | Absent |
| SA2089 | *sarR* | Staphylococcal accessory regulator R | NS |
| SA0901 | *sspA* | Cysteine protease/V8 protease | -3.8 |
| SA0900 | *sspB* | Cysteine protease precursor | -1.8 |
| SA0899 | *sspC* | Cysteine protease | -1.7 |
| SA2430 | *aur* | Zinc metalloproteinase aureolysin | -1.7 |
| SA1725 | *scpA* | Staphopain, cysteine proteinase | -9.2 |
| SA1726 | *scpB* | Hypothetical protein | -7.1 |
| SA0879 | *htrA* | Serine protease HtrA | -2.3 |
| SA0723 | *clpP* | Proteases ClpP | NS |
