# Supplementary material for: Transcriptional and Functional Analysis of the Effects of Magnolol: Inhibition of Autolysis and Biofilms in Staphylococcus aureus
Source: PLoS One. 2011 Oct 28;6(10):e26833. doi: 10.1371/journal.pone.0026833 (PMC3203910; doi:10.1371/journal.pone.0026833)
Supplement: Table S3 — The expression of genes involved in biofilm affected by MOL. a − indicates reduction and + indicates increase; NS, not significant. (DOC) [file pone.0026833.s003.doc]

| **N315 ORF** | **Gene** | **Product or putative function** | **Fold change^a^** |
| --- | --- | --- | --- |
| **Cell envelope and cellular processes** |  |  |  |
| SA2426 | *arcD* | Arginine/ornithine antiporter | 1.9 |
| SA1881 | *kdpA* | Probable potassium-transporting ATPase A chain | NS |
| SA1880 | *kdpB* | Probable potassium-transporting ATPase B chain | NS |
| SA1042 | *pyrP* | Uracil permease | -2.0 |
| SA1879 | *kdpC* | Probable potassium-transporting ATPase C chain | NS |
| SA0417 |  | Similar to sodium-dependent transporter | NS |
| SA2081 |  | Similar to urea transporter | 2.4 |
| SA1688 |  | Similar to teichoic acid translocation ATP-binding protein TagH | NS |
| SA0233 |  | PTS enzyme, maltose and glucose specific, factor II homolog | NS |
| SA0848 | *oppF* | Oligopeptide transport system ATP-binding protein homolog | 1.5 |
| SA0847 | *oppD* | Oligopeptide transport system ATP-binding protein homolog | 1.7 |
| SA0845 | *oppB* | Oligopeptide transport system permease protein | 1.5 |
| SA2242 |  | CHP (predicted permease) | 2.0 |
| SA0846 | *oppC* | Similar to oligopeptide transport system permease protein | 1.7 |
| SA0758 |  | Similar to thioredoxin | NS |
| SA2261 |  | Similar to efflux pump | NS |
| SA2132 |  | Similar to ABC transporter (ATP-binding protein) | -4.8 |
| SA0217 |  | Similar to periplasmic iron-binding protein BitC | -2.2 |
| SA1699 |  | Similar to transporter | NS |
| SA1987 | *opuD* | Glycine betaine transporter OpuD homolog | 2.2 |
| **Information pathways** |  |  |  |
| SA2424 | *acrR* | Similar to transcription regulator Crp/Fnr family protein | NS |
| SA1041 | *pyrR* | Pyrimidine operon repressor chain A | -5.9 |
| SA2320 |  | Similar to regulatory protein PfoR | -2.6 |
| SA2502 | *rnpA* | RNase P protein component | -2.4 |
| SA2134 |  | Similar to DNA 3-methyladenine glycosidase | -1.5 |
| SA0815 |  | Peptidyl-prolyl cis-trans isomerase homolog | NS |
| SA2278 |  | Similar to mutator protein MutT | -2.2 |
| SA1626 | *hsdM* | Type I restriction enzyme homolog (SaPln3) | -2.9 |
| SA0097 |  | Similar to transcription regulator AraC/XylS family | NS |
| SA2144 |  | Similar to transcriptional regulator (TetR/AcrR family) | NS |
| SA0189 | *hsdR* | Probable type I restriction enzyme restriction chain | -3.7 |
| SA1806 |  | Probable ATP-dependent helicase (bacteriophage N315) | Absent |
| **Intermediary metabolism** |  |  |  |
| SA2427 | *arcB* | Ornithine transcarbamoylase | 3.8 |
| SA2428 | *arcA* | Arginine deiminase | 4.0 |
| SA2425 | *arcC* | Carbamate kinase | 1.6 |
| SA1044 | *pyrC* | Dihydroorotase | NS |
| SA1045 | *carA* | Carbamoyl-phosphate synthase small chain | NS |
| SA1047 | *pyrF* | Orotidine-5-phosphate decarboxylase | NS |
| SA1046 | *carB* | Carbamoyl-phosphate synthase large chain | NS |
| SA2082 | *ureA* | Urease gamma subunit | 1.6 |
| SA2083 | *ureAB* | Urease beta subunit | 1.6 |
| SA1048 | *pyrE* | Orotate phosphoribosyltransferase | NS |
| SA2319 | *sdhB* | Similar to beta-subunit of L-serine dehydratase | -2.9 |
| SA2084 | *ureC* | Urease alpha subunit | 2.1 |
| SA2086 | *ureF* | Urease accessory protein | 1.9 |
| SA2088 | *ureD* | Urease accessory protein | 2.0 |
| SA2085 | *ureE* | Urease accessory protein | 2.0 |
| SA2087 | *ureG* | Urease accessory protein | 1.6 |
| SA2318 | *sdhA* | Similar to L-serine dehydratase | -2.0 |
| SA1043 | *pyrB* | Aspartate transcarbamoylase chain A | NS |
| SA2007 |  | Similar to α-acetolactate decarboxylase | -2.1 |
| SA0821 | *argH* | Argininosuccinate lyase | -1.7 |
| SA0822 | *argG* | Argininosuccinate synthase | -2.4 |
| SA2008 | *budB* | α-Acetolactate synthase | -3.9 |
| SA1155 | *cls* | Cardiolipin synthetase homolog | NS |
| SA1160 | *nuc* | Thermonuclease | -2.9 |
| SA2258 |  | Similar to diaminopimelate epimerase | Absent |
| SA1940 | *deoD* | Purine nucleoside phosphorylase | NS |
| SA1615 | *menE* | *O*-Succinylbenzoic acid-CoA ligase | NS |
| SA0925 | *purH* | Bifunctional purine biosynthesis protein | NS |
| SA0241 |  | Similar to 4-diphosphocytidyl-2C-methyl-D-erythritol synthase | 1.6 |
| SA0963 | *pyc* | Pyruvate carboxylase | NS |
| SA0011 |  | Similar to homoserine-*o*-acetyltransferase | 2.0 |
| SA0534 | *atoB* | Acetyl-CoA c-acetyltransferase | NS |
| SA0920 | *purQ* | Phosphoribosylformylglycinamidine synthase I | NS |
| SA0923 | *purM* | Phosphoribosylformylglycinamidine cyclo-ligase | NS |
| SA0924 | *purN* | Phosphoribosylglycinamide formyltransferase | NS |
| SA0242 |  | Similar to xylitol dehydrogenase | 1.5 |
| SA0921 | *purL* | Phosphoribosylformylglycinamidine synthetase | 1.7 |
| SA0922 | *purF* | Phosphoribosylpyrophosphate amidotransferase | NS |
| SA0344 | *metE* | 5-Methyltetrahydropteroyltriglutamate-homocysteine Methyltransferase | NS |
| SA0022 |  | Similar to 5'-nucleotidase | -6.7 |
| SA1814 |  | Similar to succinyl-diaminopimelate desuccinylase | 1.5 |
| SA0266 |  | CHP (ABC-type multidrug transport system, ATPase component) | -3.9 |
| **Other functions** |  |  |  |
| SA2353 | *ssaA* | Similar to secretory antigen precursor | -6.3 |
| SA0270 | *ssaA* | Similar to secretory antigen precursor | -2.1 |
| SA1629 | *splC* | Serine protease |  |
| SA0107 | *spa* | Immunoglobulin G-binding protein A precursor | 1.7 |
| **Similar to unknown proteins** |  |  |  |
| SA0023 |  | CHP | -1.6 |
| SA0814 | *kapB* | CHP | -1.9 |
| SA1692 |  | CHP (putative intracellular protease/amidase) | 1.9 |
| SA0518 |  | CHP (predicted flavoprotein) | -4.6 |
| SA1612 |  | CHP (NTP pyrophosphohydrolases) | -2.4 |
| SA1133 |  | CHP | -1.6 |
| SA2371 |  | CHP | NS |
| SA0559 |  | CHP (histone acetyltransferase HPA2 and related acetyltransferases) | NS |
| SA0872 |  | CHP (enterochelin esterase and related enzymes) | -1.9 |
| SA2131 |  | CHP (ABC-type Na^+^ efflux pump, permease component) | -2.3 |
| SA1733 |  | CHP | NS |
| SA2322 |  | CHP (permeases of the drug/metabolite transporter superfamily) | -1.6 |
| SA0269 |  | HP | -10.0 |
| SA0359 |  | CHP (uncharacterized membrane protein) | 1.7 |
| **No similarity** |  |  |  |
| SA1049 |  | HP | NS |
| SA0575 |  | HP | Absent |
| SA1152 |  | HP | -1.8 |
| SA0752 |  | HP | 1.8 |
| SAS025 |  | HP | NS |
| SA2372 |  | HP | NS |
| SA0364 |  | HP | -3.5 |
| SA1332 |  | HP | NS |
| SA1015 |  | HP | -1.8 |
| SA2373 |  | HP | NS |
| SA0740 |  | HP | 1.8 |
| SA0268 |  | HP | -12.5 |
| SA0267 |  | HP | -5.9 |
| SA1726 |  | HP | -7.1 |
