# Supplementary material for: Transcriptional and Functional Analysis of the Effects of Magnolol: Inhibition of Autolysis and Biofilms in Staphylococcus aureus
Source: PLoS One. 2011 Oct 28;6(10):e26833. doi: 10.1371/journal.pone.0026833 (PMC3203910; doi:10.1371/journal.pone.0026833)
Supplement: Table S4 — Primers used in real-time RT-PCR with SYBR green probes. a ORF, open reading frame. (DOC) [file pone.0026833.s004.doc]

| **Primer** | **N315 ORF*^a^*** | **Sequence** |
| --- | --- | --- |
| 16S rRNA for | SArRNA01 | CGTGCTACAATGGACAATACAAA |
| 16S rRNA rev | SArRNA01 | ATCTACGATTACTAGCGATTCCA |
| atl for | N315-SA0905 | TACCGTAACGGCGTAGGTCGT |
| atl rev | N315-SA0905 | CATAGTCGTGTGTGTGTACGA |
| Sle1for | N315-SA0423 | GTAGCCGTCCATCAACGAACT |
| Sle1 rev | N315-SA0423 | CTATTGCTCGCAGCGTTACT |
| cidA for | N315-SA2329 | CTTAGCCGGCAGTATTGTTG |
| cidA rev | N315-SA2329 | TGAAGATAATGCAACGATAC |
| lytM for | N315-SA0265 | ATGCCAATGGAAGCGGCCA |
| lytM rev | N315-SA0265 | TTCGCATGACCACTAGCTGT |
| lytN for | N315-SA1090 | GGAGACACACTTAGTGCT |
| lytN rev | N315-SA1090 | CTAATGGTGTCATTGGCACT |
| lrgA for | N315-SA0252 | CTGGTGCTGTTAAGTTAGGCG |
| lrgA rev | N315-SA0252 | GTGACATAGCCAGTACAAAT |
| lrgB for | N315-SA0253 | CGGTACAGTTGTAGCGTTATTA |
| lrgB rev | N315-SA0253 | AGTGCTAATCCTCGGGCAATA |
| arlR for | N315-SA1248 | GCGCTTAGCCATTACTATGAT |
| arlR rev | N315-SA1248 | ATCGTCTGCACCGTAATCAAGC |
| sarA for | N315-SA0573 | CGCTGTATTGACATACATCAGCG |
| sarA rev | N315-SA0573 | CTCGACTCAATAATGATTCGA |
| cidB for | N315-SA2328 | GACGTCATTGTAACGTTATTGC |
| cidB rev | N315-SA2328 | TGAACTAAATGCACCGGATTC |
| cidC for | N315-SA2327 | GAGACTCAATCGACGCAGTTGT |
| cidC rev | N315-SA2327 | TCCAAGTGCTGTACTATTCG |
| agrA for | N315-SA1844 | TGAAATTCGTAAGCATGACCC |
| agrA rev | N315-SA1844 | CATCGCTGCAACTTTGTAGAC |
| RNAⅢ for | N315-SAS065 | ATGAGTTGTTTAATTTTAAGAAT |
| RNAⅢ rev | N315-SAS065 | CACTGTGTCGATAATCCA |
| lytR for | N315-SA0251 | CTGCACATGACCAATACGC |
| lytR rev | N315-SA0251 | TCGCCGACATATCATTCGCA |
| lytS for | N315-SA0250 | GCATATGCGGTTGACCTCATAT |
| lytS rev | N315-SA0250 | GCTTGTAATTGCTACGGCAGA |
| mgrA for | N315-SA0641 | CAAGTTAATCGCTACTACTC |
| mgrA rev | N315-SA0641 | CTTCACGTTGATCGACTTCG |
| arlS for | N315-SA1246 | GTATATCATTGCGCTGGCA |
| arlS rev | N315-SA1246 | GTGTTCGTAATTCATGTGACGC |
| icaA for | N315-SA2459 | CTGGCGCAGTCAATACTATTTCGGGTGTCT |
| icaA for | N315-SA2459 | GACCTCCCAATGTTTCTGGAACCAACATCC |
| icaC for | N315-SA2462 | TGCGTTAGCAAATGGAGACTATTG |
| icaC rev | N315-SA2462 | TGAGAAAGCACTAATCATTTGAATCG |
